# Molecular foundations of chilling-tolerance of modern maize

**DOI:** 10.1186/s12864-016-2453-4

**Published:** 2016-02-20

**Authors:** Alicja Sobkowiak, Maciej Jończyk, Józef Adamczyk, Jarosław Szczepanik, Danuta Solecka, Iwona Kuciara, Katarzyna Hetmańczyk, Joanna Trzcinska-Danielewicz, Marcin Grzybowski, Marek Skoneczny, Jan Fronk, Paweł Sowiński

**Affiliations:** Department of Plant Molecular Ecophysiology, Institute of Plant Experimental Biology and Biotechnology, Faculty of Biology, University of Warsaw, Miecznikowa 1, 02-096 Warszawa, Poland; Plant Breeding Smolice Co. Ltd., Smolice 146, 63-740 Kobylin, Poland; Department of Molecular Biology, Institute of Biochemistry, Faculty of Biology, University of Warsaw, Miecznikowa 1, 02-096 Warszawa, Poland; Department of Genetics, Institute of Biochemistry and Biophysics, Polish Academy of Sciences, Pawińskiego 5A, 02-106 Warszawa, Poland; Present address: Department of Genetics, Institute of Psychiatry and Neurology in Warsaw, Sobieskiego 9, 02-957 Warsaw, Poland

**Keywords:** Acclimatization, Cell walls, Cold-sensitivity, Maize, Microarrays, miRNA, Photosynthesis, Seedling

## Abstract

**Background:**

Recent progress in selective breeding of maize (*Zea mays* L.) towards adaptation to temperate climate has allowed the production of inbred lines withstanding cold springs with temperatures below 8 °C or even close to 0 °C, indicating that despite its tropical origins maize is not inherently cold-sensitive.

**Results:**

Here we studied the acclimatory response of three maize inbred lines of contrasting cold-sensitivity selected basing on multi-year routine field data. The field observations were confirmed in the growth chamber. Under controlled conditions the damage to the photosynthetic apparatus due to severe cold treatment was the least in the cold-tolerant line provided that it had been subjected to prior moderate chilling, i.e., acclimation. The cold-sensitive lines performed equally poorly with or without acclimation. To uncover the molecular basis of the attained cold-acclimatability we performed comparative transcriptome profiling of the response of the lines to the cold during acclimation phase by means of microarrays with a statistical and bioinformatic data analysis.

**Conclusions:**

The analyses indicated three mechanisms likely responsible for the cold-tolerance: acclimation-dependent modification of the photosynthetic apparatus, cell wall properties, and developmental processes. Those conclusions supported the observed acclimation of photosynthesis to severe cold at moderate chilling and were further confirmed by experimentally showing specific modification of cell wall properties and repression of selected miRNA species, general regulators of development, in the cold-tolerant line subjected to cold stress.

**Electronic supplementary material:**

The online version of this article (doi:10.1186/s12864-016-2453-4) contains supplementary material, which is available to authorized users.

## Background

Maize is a major crop plant cultivated all around the world except for the far northern and southern regions of the Earth. Its economic importance has encouraged multifarious studies, including those on its complex genetics, physiology, and domestication. Notably, maize is also a model plant of growing popularity [1], the first C4 crop with a sequenced genome [2]. From the botanical point of view, several maize subspecies are known, the most important being *Zea mays* spp*. indentata, Z. mays* spp*. indurata, Z. mays* spp*. saccharata* and *Z. mays* spp. *everta*. For the breeding practice, however, more important are germplasm lineages represented by thousands of inbred lines used for production of hybrids in the public and private sectors. The strong physiological diversity of the inbreds reflects substantial differences at the molecular level. Two lines may differ in the expression pattern of hundreds of genes [3] and their genomes may be less related to each other than are those of humans and the chimpanzee [4]. The intraspecific diversity of maize underlies its excellent adaptability to contrasting climates. This feature was used by ancient farmers during maize domestication and is still being exploited by modern breeders for improving different traits of the crop. An important example is the breakdown of the photoperiodic requirements allowing the early (pre-Columbian) poleward shift of maize cultivation in the Americas relative to the site of its origin, Mexico, and the improvement of drought tolerance crucial for the adaptation to the North American conditions [5]. Another, more recent example is the ongoing gradual adaptation of maize to the temperate climate with cold springs and short summers.

The most important factor limiting maize adaptation to the conditions prevalent at higher latitudes was its cold-sensitivity manifested by strong retardation of growth and development at temperatures below 17 °C, severe injuries below 8 °C, and even death below 4 °C. The mechanisms underlying the maize cold-sensitivity have been studied for many years [6]. At the physiological level the research concerned mostly the photosynthetic apparatus in respect to both the light and the dark phase (ibid.), diverse aspects of root functioning [7], water relations [8], and transport processes [9]. In this respect, one should consider the ability of maize to acclimate to the cold. Acclimation is defined as an increase of tolerance of severe stress by weak stress [10]. Indeed, maize seems capable of cold-acclimation as its growth at moderately low temperatures (10 -14 °C) has been shown to improve the tolerance of severe cold. Diverse processes related to the maize reactions to low temperatures have been shown to be modified upon acclimation. The reactions concerned improvement of the photosynthetic apparatus efficiency in the cold [11], changes in photosynthetic enzymes [12], and the activities of non-enzymatic [13] and enzymatic [14] antioxidant systems. Also the metabolite exchange path between photosynthetic and non-photosynthetic cells responsible for exporting assimilates from the leaf was found to respond favourably to cold acclimation [15]. Others showed that water balance could be improved by acclimation due to changes in membrane properties [8]. One should note, however, that acclimation improves the maize cold-tolerance only marginally, by a few degree Celsius, and does not resemble the hardening of cold-resistant plants such as *A. thaliana* leading to frost tolerance [16, 17].

At the molecular level, several genes potentially linked to the maize response to the cold have been identified. Among them were genes related to photosynthesis, sugar metabolism, and secondary metabolism [18–22]. Transcriptome profiling suggested a role of genes related to the circadian rhythm and the cell membrane/cell wall system [23, 24].

Maize orthologs of genes engaged in cold-signal transduction in cold-resistant plants, such as *ZmCDPK1* [25] and *ZmDREB1A* and *ZmDREB2A* [26] from the ERF/AP2 (ETHYLENE RESPONSE FACTOR/APETALA2) family have also been implicated. ERF/AP2 family transcription factors induce expression of numerous *cor* (cold regulated) genes in *A. thaliana* [17]. Our transcriptomic data [23, 24] confirmed cold-dependent induction of several *DREB* genes, but found no expression changes for orthologs of other cold-regulated genes from cold-tolerant plants, such as *CBF* (C-repeat binding factor) [27]. Thus, despite some advances, the molecular basis of maize cold-sensitivity is still far from being fully understood, particularly in the context of its cold-acclimation.

The main reason for this unsatisfactory progress is a lack of a suitable model system. The most fruitful approach would initially involve a direct comparison, by diverse techniques, of maize variants showing markedly different reactions to low temperature. To the best of our knowledge, however, no maize materials expressing cold-resistance have been reported, making such a comparative study impossible until now. Recently, while carrying out field observations as part of a routine breeding program (not specifically addressing cold-tolerance) we came across a very promising inbred line showing remarkable early vigor despite a cold spring with average temperatures well below 8 °C and even sub-zero at nights, calling into question the popular belief that maize, similarly to other crops of tropical origins, is inherently cold-sensitive.

Here we used this line to unravel the molecular basis of the acquired cold-tolerance using transcriptomics supported by physiological assessments. The working hypothesis was that the trait is related to the plant’s ability to acclimate to the cold (below 8 °C) at moderately low temperatures (12 -15 °C), expressed both at the physiological and molecular levels. For comparison with the cold-tolerant S68911 line we used two cold-sensitive ones, S160 and S50676. S160 has been studied earlier and shows extreme cold-sensitivity [28], in this respect constituting a perfect counterpart to S68911. However, the two lines derive from distant gene pools [29], which could complicate the interpretation of transcriptomic data. To avoid this problem we introduced the second cold-sensitive reference line, S50676, from the same pedigree as the cold-tolerant one, S68911.

## Results

### Field and physiological characteristics of maize inbred lines

The plant material for the transcriptomic studies addressing the molecular mechanism of maize tolerance to low temperature was selected using data collected under both field and controlled growth conditions. Several inbred lines were tested for their performance under field conditions in the West Poland region by assessing their early vigor estimated in a 1-9 scale (1 the lowest, 9 the highest) at the stage of the fourth leaf, and the effective temperature sum (ETS) from sowing to 50 % of silking (Additional file 1). In parallel, plants grown under controlled conditions were evaluated visually as well as by measuring the efficiency of the photosynthetic apparatus at low temperatures. Those assessments combined identified three inbred lines of a contrasting low-temperature tolerance: the cold-tolerant S68911 and two lines of moderate-to-high cold-sensitivity, S50676 and S160. S68911 and S50676 were of a highly similar genetic background (Stiff Stalk Synthetic/Iodent), while S160 was distant from those two (pedigree 354).

**Fig. 1:**
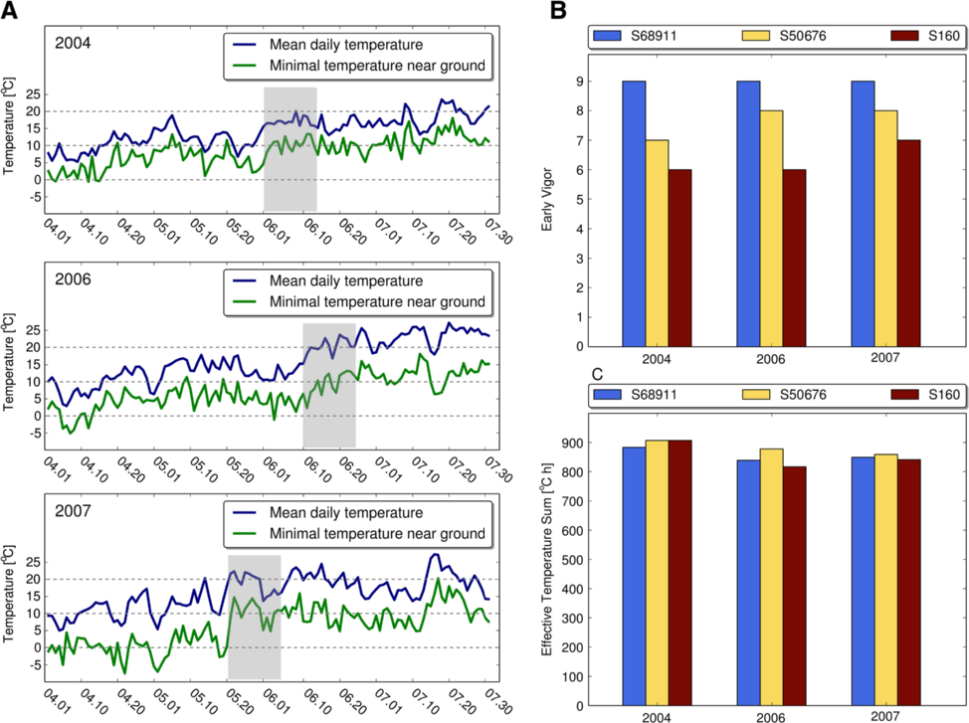
Field parameters of three maize inbred lines at Smolice location. **a** Daily temperatures in years 2004, 2006, 2007; shadowing indicates time of early vigor evaluation; **b**. Early Vigor; **c**. Effective Temperature Sum

Results of the field observations, including local average daily air temperatures and minimal temperatures at ground level, are shown in Fig. 1. Of the three years taken into consideration, one (2004) had an extremely cold spring. Daily maximal and minimal temperatures in those years are shown in Additional file 2. The S68911 line consistently showed the maximal early vigor, which reflected the perfect performance of its seedlings even under the cold 2004 spring conditions. At the other extreme was the S160 line showing consistently the weakest early vigor, even in the relatively warm 2007 year. The S68911 line outperformed the other lines also in respect to the development rate until the generative phase in the cold year, as reflected by its lowest ETS value. In the two warmer years, however, the picture was slightly different and the lowest ETS was noted for the S160 inbred line, S68911 being a close second. Taken together, the field data clearly show the superiority of the S68911 line over S50676, and especially S160, under cold spring conditions.

**Fig. 2:**
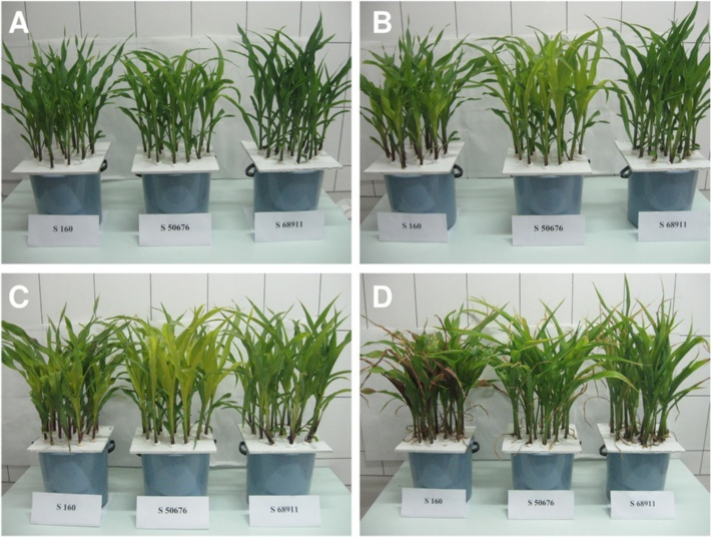
Seedling performance at various temperatures. Seedlings of three inbred lines were grown to V3 stage at 24 °C/22 °C (**a**), transferred for four days to 14 °C/12 °C (**b**), then for four days to 8 °C/6 °C (**c**), and finally for two days to 24 °C/22 °C (**d**). Photographs were taken at the end of each period

**Fig. 3:**
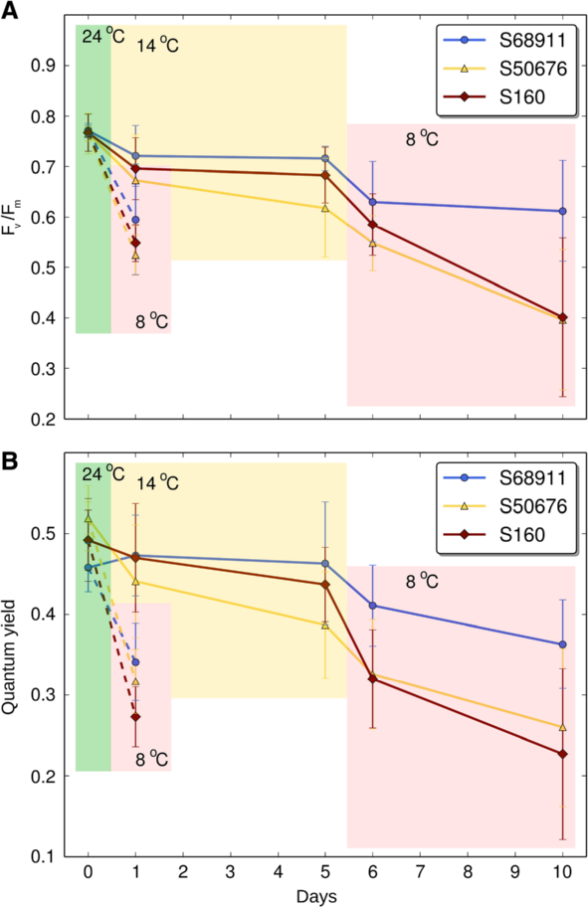
Maximum quantum efficiency of PSII primary photochemistry and effective quantum yield of PS II. Fv/Fm (**a**) and ΦPSII (**b**) were measured in the 3rd leaf of plants of three maize inbred lines grown at 24 °C/22 °C (control, day 0, green background), then the plants were transferred to 14 °C/12 °C for four days (acclimation, days 1 and 5, yellow background), and finally to 8 °C/6 °C for four days (severe cold, days 6 and 10, pink background). Alternatively, plants were transferred directly from 24 °C/22 °C to 8 °C/6 °C (pink background, broken lines). Data are means ± SD for three independent experiments with 3 - 5 plants per experiment

We next checked if the differences in the cold-tolerance of the three maize lines observed in the field were also expressed under controlled conditions in which plants for the key transcriptomic part of this study were to be grown. Since the study was focused on the ability of maize to acclimate to cold stress, plants at the third-leaf stage were first chilled for four days at 14 °C/12 °C (acclimation) and then cooled for another four days at 8 °C/6 °C (severe cold stress). As shown in Fig. 2, only the S68911 seedlings showed no symptoms of injury by the severe cold. S50676 showed many drying leaf ends, while the S160 plants had whole leaves injured and drying and some apparently dead. Additionally, basic chlorophyll fluorescence parameters (maximum quantum efficiency of PSII primary photochemistry, Fv/Fm, and effective quantum yield of PSII electron transport, Φ_PSII_) were determined to gauge the degree of the chilling-induced injury to the photosynthetic apparatus. Figure 3 shows data on the both parameters. It is evident that the photosynthetic apparatus efficiency of the S68911 seedlings was higher than that of the other lines tested throughout the experiment, but especially so during the severe cold stress. To check if the latter effect depended on acclimation of the seedlings, they were transferred from the optimal conditions directly to the severe cold, without the acclimation at 14 °C/12 °C. Unlike in the previous experiment, here all three lines showed similar Fv/Fm and Φ_PSII_ values upon cold treatment, thus demonstrating the importance of the acclimation period for protecting the photosynthetic apparatus of S68911 against the effects of severe cold stress. All in all, the reported set of experiments carried out in controlled conditions confirmed the field-study results showing the best cold-performance of the S68911 line and the highest cold-sensitivity of S160, and underscored the critical role of acclimation.

### Microarray experiment

To study the molecular basis of the presented differences in cold tolerance, we analyzed transcriptome-level response of the three inbred lines to low temperature. Since we observed a substantial acclimatory effect on the photosynthetic apparatus after four days of moderate cold treatment, we used roughly half of that period (14 °C/12 °C for 38 h) to study the gene expression changes.

The transcriptomic response of all investigates lines to the chilling treatment was pronounced. With the false discovery rate correction set at 0.05 (*p*-value = 0.0054), as many as 12,856 probes (that is, *ca.* 30 % of all) showed a significant change of signal strength in at least one line. Even with a more stringent false discovery rate correction, *p* = 0.01 (*p*-value = 0.00058), the number of responding probes - 7818 – was still substantial. The genes responding to the chilling were categorized using the Gene Ontology system and assigned a putative function using various approaches, including advanced data mining, and differences and similarities in the response of the lines were determined for the genes with a meaningful description. In all analyses probe redundancy was eliminated, *i.e.*, when several microarray probes matched a single gene it was counted only once.

The microarray data were verified by quantitative RT-PCR. To this end, 29 genes representing different signal strengths were selected. Primers used are shown in Additional file 3. The *GAPDH* transcript was used as a reference with a steady level of expression. The results of the RT-qPCR analysis are shown in Additional file 4 together with corresponding microarray data. The correlation coefficients between the data obtained with these two methods are 0.93, 0.94, and 0.93 for the S68911, S50676 and S160 inbred lines, respectively. Thus, the microarray results of this study faithfully represent the true abundances of individual transcripts.

### Global analysis of microarray data

A global analysis of the chilling-responsive genes with the Gene Ontology system was performed to identify over-represented (enriched) GO categories in each of the lines individually. Here, sets of genes selected with the more stringent FDR of 0.01 were used to avoid distortion of the results by genes showing minor expression change. Raw results of those analyses are presented in Additional files 5, 6, 7, 8, 9, 10, 11, 12, 13, 14, 15, 16, 17, 18, 19, 20 and 21 showing enriched GO categories among the chilling-induced (up-regulated) or repressed (down-regulated) genes for each line and each GO class (Molecular Function, Cellular Component and Biological process) separately. For an analysis of the similarities and differences among the inbred lines in their general response to the chilling only the most informative (the lowest-level) over-represented GO classes shown in Tables 1 (Molecular Function), 2 (Cellular Component), and 3 (Biological Process) were used.

**Table 1:**
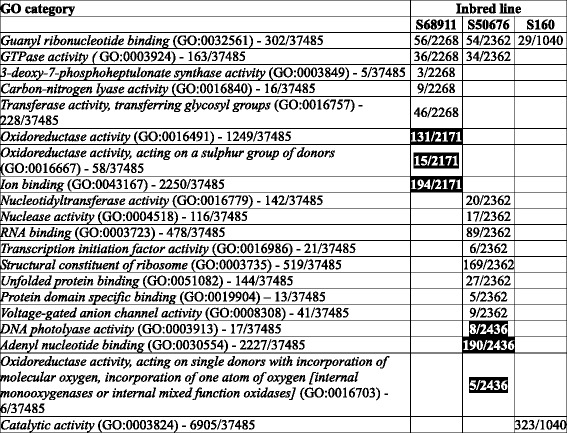
Gene Ontology categories in Molecular Function class significantly over-represented among transcripts affected by cold treatment in three maize inbred lines

Numbers in column “GO category” indicate total number of genes described by given GO term/size of the population (total number of GO-assigned genes). Numbers in columns “Inbred line” indicate number of cold-affected genes described by the GO term in a given line/total number of cold-affected genes. Induction of expression is in normal font, repression in white font on black background

**Table 2:**
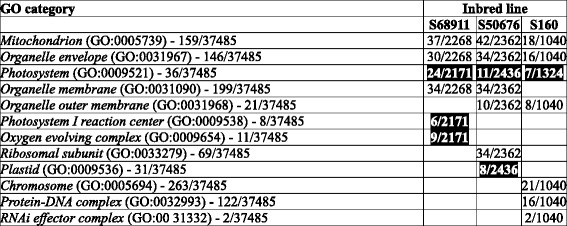
Gene Ontology categories in Cellular Component class significantly over-represented among transcripts affected by cold treatment in three maize inbred lines. Other descriptions as in Table 1

**Table 3:**
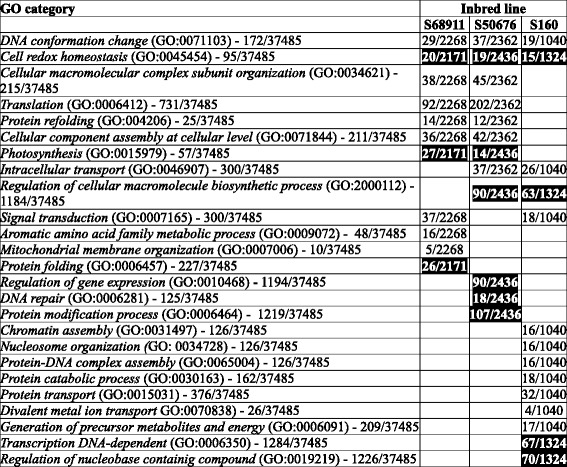
Gene Ontology categories in Biological Process class significantly over-represented among transcripts affected by cold treatment in three maize inbred lines. Other descriptions as in Table 1

In the GO class “Molecular Function”, apart from only two enriched categories common to all three or two of the lines studied (Table 1), as many as 18 categories were unique to one line only: six specific for S68911 (three induced and three repressed), eleven for S50676 (eight induced and three repressed) and one induced specific for S160. This distribution highlights the profound molecular differences among the lines studied, but is difficult to interpret since the GO categories in this class cannot be unambiguously connected with any particular physiological process.

In the GO class “Cellular Component” (Table 2), unlike in the previous one, the overall response seemed remarkably uniform across lines, showing that despite the physiological and molecular differences the cellular components most significantly affected by chilling were the same in all three maize lines studied. Genes related to Mitochondrion (GO:0005739) and Organelle envelope (GO:0031967) were up-regulated and those related to Photosystem (GO:0009521) down-regulated. A few categories were unique to a single line, most notably three up-regulated ones in the cold-sensitive S160 line related to chromatin structure and functioning.

The results concerning GO class “Biological Process” (Table 3) reflected the relations among the lines studied. The response common to all three lines comprised up-regulation of genes related to DNA conformation change (GO:0071103) and down-regulation of those related to Cell redox homeostasis (GO:0045454). The two genetically close lines, S68911 and S50676, also showed marked similarity in their transcriptomic response, sharing with each other five enriched categories, including four up-regulated ones related to protein synthesis and assembly; only three categories were unique to each of the two lines. In contrast, the genetically more distant S160 line showed a unique transcriptomic response in nine categories, most notably the up-regulation of genes related to chromatin assembly and protein catabolism, and down-regulation of those related to transcription and nucleic acid metabolism.

### Detailed analysis of microarray data

To add physiological relevance to the observed differences in the transcriptomic responses of the three lines studied, highlighted especially by the GO analysis in the Molecular Function class, a detailed gene-by-gene analysis was carried out. To select the genes of potential interest, those showing significant (FDR = 0.05) expression changes in at least one inbred were clustered into 30 sets according to the magnitude of their expression change among all three maize lines, and clusters comprising genes changing their expression exclusively in a single inbred were subjected to further analysis. Those genes were inspected for the functions they could play in the response to chilling. Functional annotations of the genes were based on an extensive manual literature search using traditional search engines (PubMed, Scopus, Google, etc.) and software-aided literature data-mining with the use of Pathway Studio 9.0.

**Fig. 4:**
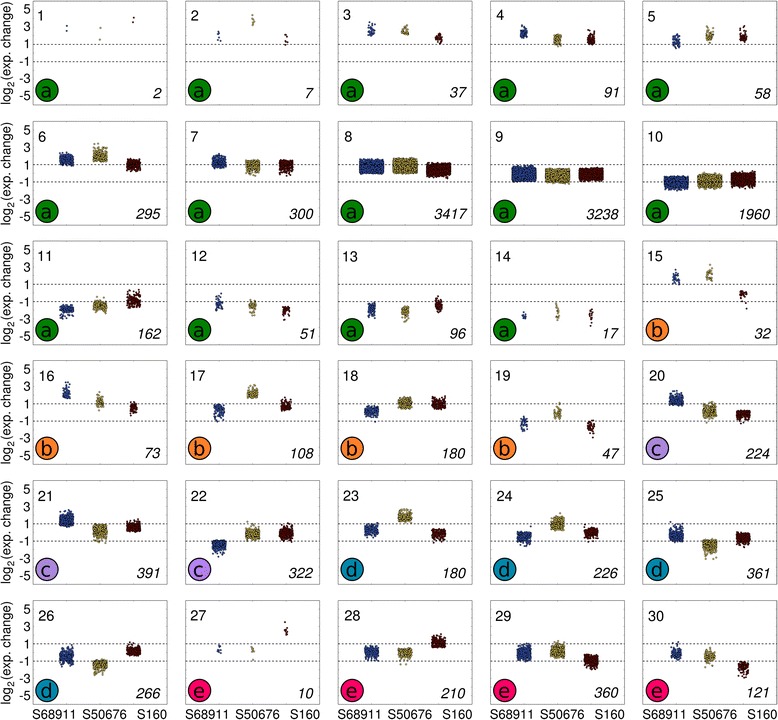
Clustering of genes according to magnitude of expression change upon cold treatment. Clusters were grouped manually into five sets (a – e) basing on the similarity of response in the three inbred lines studied. a. clusters 1-14, genes showing similar response in all three lines, from strongly induced to strongly repressed at low temperature, b. clusters 15-19, genes showing similar expression changes in two of the lines and no or small changes in the third line. c. clusters 20-22, genes specific to line S68911 (specific genes are defined as genes changing markedly - median magnitude of change in a cluster ≥2 – in a single line only), d. clusters 23-26, genes specific to line S50676. e. clusters 27-30, genes specific to line S160. Gene expression level was estimated in 3rd leaves of plants of three maize inbred lines treated with 14 °C/12 °C for 38 h (dark period + light period + dark period + 4 h of light period) in relation to the level in control plants grown at 24 °C/22 °C. The ratio of expression levels (ordinate) is shown as log_2_(cold/control). Inbred lines are indicated at the bottom of figure (abscissa). Each data point corresponds to a single gene; the points coalesce in densely populated regions. Cluster number is shown in left-hand top corner of each plot, cluster set in left-hand bottom corner, number of genes in a given cluster in right-hand bottom corner

As presented in Fig. 4, almost 80 % of genes showed similar expression changes in all three maize lines. These genes grouped in clusters 1-14 forming a gradient of the magnitude of expression change, from strongly induced (cluster 1) to strongly repressed (cluster 14) at low temperature. Clusters 15-19 comprised genes showing similar expression changes in two of the lines and no or small changes in the third line. Finally, clusters 20-30, grouping altogether 2672 genes, were subjected to a further analysis as they comprised genes changing markedly (median magnitude of change in each cluster ≥2) in a single line only. Thus, clusters 20-22 were specific to the S68911 line, 23-26 to S50676, and clusters 27-30 to S160.

**Fig. 5:**
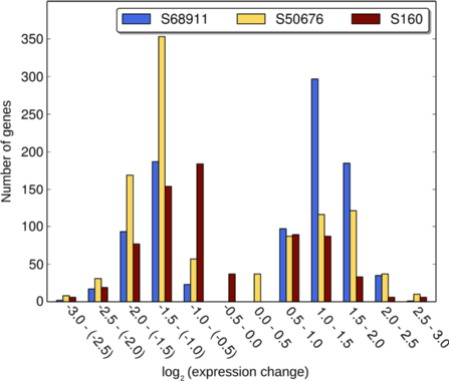
Distribution of specific genes according to magnitude of expression change upon cold treatment. Specific genes are those showing up-regulation or down-regulation in a single inbred line only and no change in other two inbred lines. The ratio of expression levels is shown as log2(cold/control)

**Fig. 6:**
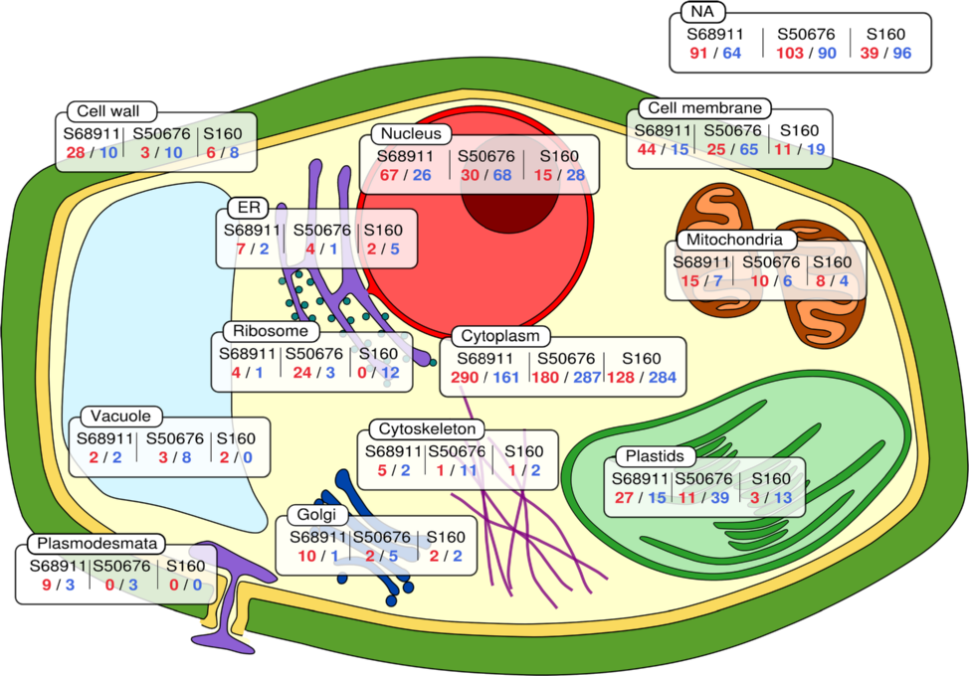
Predicted cellular localizations of products of specific genes. Specific genes are those showing up-regulation or down-regulation upon cold treatment in a single inbred line only and no change in other two inbred lines. Localization of proteins was assigned basing on GO annotation (Cellular Component) or InterPro domain description. Rectangles show the cell compartment and the numbers of up-regulated (red)/down-regulated genes (blue) in each inbred line. Proteins assigned to Cytoplasm include also those with a known annotation but unknown cytolocalization. Rectangle marked NA represents probes lacking annotation

In respect to the line-specific cold-responsive genes the cold-tolerant S68911 line was clearly distinct from the two others. The major difference was that gene up-regulation was the dominant response to the cold in S68911 (induced:repressed genes, *ca.* 2:1) and down-regulation – in the two other lines (induced:repressed genes, *ca.*1:2) (Fig. 5). This general difference was also well-pronounced when we considered separately, as a first step towards elucidation of their role in cold response, sets of genes whose products were related functionally to particular cellular compartments, organelles or structures (Fig. 6) and were related to particular processes (Additional files 22, 23 and 24). The prevalence of induction over repression in S68911 was particularly strong for genes related to the cell structures engaged in the communication with the surroundings: cell wall, cell membrane, plasmodesmata, and Golgi apparatus. The same was true for the related processes of transport (up-regulated/down-regulated genes, 26/11 in S68911, 12/33 in S50676, and 6/13 in S160), cell wall modification (20/0, 2/7, and 4/3), and signal transduction (28/10, 7/21, and 1/1). Also genes related to the chloroplasts, showed the same clear-cut tendency (Fig. 6), although, as an exception, for genes related to the redox balance it was much less obvious (19/13, 12/12, and 8/12).

The most numerous were genes related to the nucleus (Fig. 6), including many encoding transcription factors and here as well the contrast between S68911 and the two cold-sensitive lines was remarkable, suggesting massive downstream modulation of transcription in that cold-tolerant line. This was reflected at the process level, with strong S68911-specific up-regulation of protein modification (up-regulated/down-regulated in S68911, S50676, and S160 line, respectively: 24/15, 20/28, and 9/15 genes), lipid metabolism (21/8, 4/7, and 2/4), and carbohydrate metabolism (18/5, 4/22, and 3/5), all processes possibly involved in cold-stress acclimation. Most impressive were the differences in the effect of cold on genes related to cell cycle and development (25/10, 9/12, and 7/12). One should note that among the relatively few cold-repressed genes in S68911 is *DCL1* whose product is crucial for miRNA maturation [30] and, in turn, miRNAs are generally negative regulators of developmental processes. It could be therefore concluded that the prevalent up-regulation of gene expression observed in the cold-tolerant S68911 at low temperature reflects induction of acclimatory metabolic and developmental changes in this line, as opposed to the S50676 and S160 lines.

In contrast to the cold-tolerant S68911 line, the two others showed little specificity in their transcriptomic response to the cold. The only marked peculiarities were the dominance of induction over repression for dozens of genes related to ribosomes in the S50676 line, suggesting induction of translation, and the general character of the response of the S160 line. The number of cold-responsive genes specific to this line (with probe redundancy deleted) was substantially lower than in the two other lines (692 in S160 *vs.* 909 in S68911 and 995 in S50676), and also the magnitude of their expression change was small in general. Thus, the transcriptomic response of the most cold-sensitive line, S160, is substantially less pronounced – both concerning its range and strength – than that of the two other lines studied.

### Unique genes

Since maize exhibits exceptionally high intraspecific genome sequence diversity, we reasoned that one of the causes of the diverse cold-sensitivity of the inbred lines could lie in differences in the basal expression levels of a handful of individual genes among those lines. Therefore we performed a comprehensive analysis of the microarray data to find such genes that we dubbed “unique” and defined them as those expressed under non-stressed growth conditions at a level substantially greater than the microarray negative control (>6 arbitrary log_2_ units of fluorescence intensity) in a single maize line and showing a statistically significant (as defined earlier) response to cold, while being expressed at a level close to or below that of the negative control in the two other lines. Four such genes were found, all expressed specifically in the S68911 line and all showing repression at low temperature. We verified their expression levels using RT-qPCR (Table 4). The table also gives putative characteristics of those genes.

**Table 4:** Characteristics of unique genes

Probe ID (MZxxxxxxxx) Gene ID (GRMZM2Gxxxxxx) Annotations (MaizeGDB)	Gene expression in control conditions by microarrays [log_2_(control)]			Gene expression in control conditions by RT-qPCR 2^(ctk-ctK)^			Cold-induced gene expression change by microarrays [log_2_(c/k)]			Cold-induced gene expression change by RT-qPCR [ΔΔ(CT)]		
I	II			III			IV			V		
	S68911	S50676	S160	S68911	S50676	S160	S68911	S50676	S160	S68911	S50676	S160
MZ00021278; GRMZM2G019907 MaizeCyc: Ocs-element binding factor 3. BP: regulation of transcription, DNA-dependent CC: nucleus	8.14	6.88	6.85	0.0032 ± 0.0018	0.0014 ± 0.0008	0.0012 ± 0.0003	-2.1^a^	-0.88 ns	-0.8 ns	-1.98 ± 0.67	-0.71 ± 0.29	-0.63 ± 0.05
MZ00026395; GRMZM2G331566 MaizeCyc: cellulase AT4G02290.1: glycosyl hydrolase 9B13 CC: extracellular region	9.29	5.85	5.49	0.0417 ± 0.0173	0.0113 ± 0.0056	0.0006 ± 0.0002	-1.63^a^	-0.69 ns	-0.38 ns	-1.64 ± 0.15	-0.83 ± 0.47	-0.33 ± 1.28
MZ00029516; GRMZM2G108576 AT3G06260.1(GATL4): galacturonosyltransferase-like 4 CC: Golgi apparatus	8.38	6.64	5.14	0.2713 ± 0.1	0.0783 ± 0.056	0.0454 ± 0.0105	-1.37^a^	-1.5^a^	-0.47 ns	-1.26 ± 0.45	-1.54 ± 2.49	-0.88 ± 0.07
MZ00056664; GRMZM2G176998 Gramene Annotations: Putative WD40-like beta propeller repeat family protein AT1G21680.1: DPP6 N-terminal domain-like protein CC: plant-type cell wall, extracellular region, vacuole	10.5	6.67	6.18	0.2413 ± 0.1477	0.009 ± 0.001	0.0034 ± 0.0025	-2.32^a^	0.05 ns	-0.68 ns	-1.78 ± 1.07	0.28 ± 0.09	-0.72 ± 0.2

Column I gives microarray probe ID, gene ID according to Maize Sequence Project, and annotations according to MaizeGDB and related databases (MaizeCyc, Gramene). Column II gives transcript level in control plants estimated by microarray hybridization; value close to 6 or below indicates no expression. Column III gives transcript level in control plants estimated by RT-qPCR in relation to reference gene (*GAPDH*). Column IV presents expression change (cold, c *vs* control, k) as found by means of microarrays; data are means of 4 biological experiments; ^a^ marks statistical significance at false discovery rate correction set at 0.05 (*p*-value = 0.0054). Column V presents expression change (cold *vs* control) as found by means of RT-qPCR; data are means of 4 biological experiments (with 3 technical replications) ± SD

To find the reason for their contrasting expression levels among the maize lines, we sequenced putative promoter regions - defined arbitrarily as *ca.* 1000 bp upstream from the predicted translation start codon - of those four genes from the three inbred lines studied. Three of the genes showed either identical promoter sequences in all three lines (the genes represented by probes MZ00056664 and MZ00021278), or only minor sequence alterations possibly reflecting the genetic relatedness of the lines but unrelated to their levels of the gene’s expression (MZ00029516, results not shown). The GRMZM2G331566 gene (represented by probe MZ00026395) encoding a cellulase, however, showed a clearly different pattern. Remarkably, its putative promoter sequence from the cold-tolerant S68911 line differed substantially from those from the two cold-sensitive lines, where the promoters were identical to each other. Those differences comprised a large insertion of 132 nucleotides and a deletion of 24 nucleotides, several small indels, and a number of point mutations (Additional file 25). A search for putative transcription-factor-binding sequences in the two variants of the GRMZM2G331566 upstream region revealed, in addition to numerous common motifs, two unique to the S68911 variant recognized by the MYB15 and MYB84 factors, respectively, and one, recognized by bZIP911, absent in the S68911 variant but present in the two other lines. These results indicate that the observed differences in the putative promoter sequence between the S68911 and the two other lines could be responsible for the highly divergent pattern of GRMZM2G331566 expression in the lines studied. Experimental verification of this conjecture is under way.

### Cell wall properties and quantitation of selected miRNAs

To verify the main conclusions emerging from the transcriptomic studies, *i.e.*, the major role of the cell wall in the acclimation of the cold-tolerant S68911 line and possible induction of developmental processes in that line, we studied those aspects of plant functioning directly. To check if the expected changes have a transient nature and represent the alarm phase of stress response [10] or are long-lasting, constituting part of the acclimation phase (*ibid.*), we extended the period of cold-treatment to one week and took samples every other day.

**Fig. 7:**
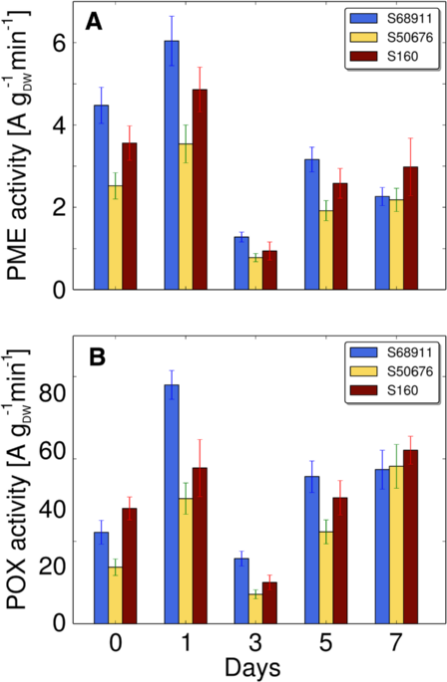
Changes in activity of cell wall enzymes upon cold treatment. Plants of three maize inbred lines were grown at 24 °C/22 °C (control, day 0) and transferred to 14 °C/12 °C for seven days. Crude cell wall was prepared from 3rd leaves at time points indicated and enzyme activities were measured as described in Materials and Methods. **a** Pectin methylesterase activity (PME); **b**. Peroxidase activity (POX). Data are means ± SD for three independent experiments with three plants per experiment

**Fig. 8:**
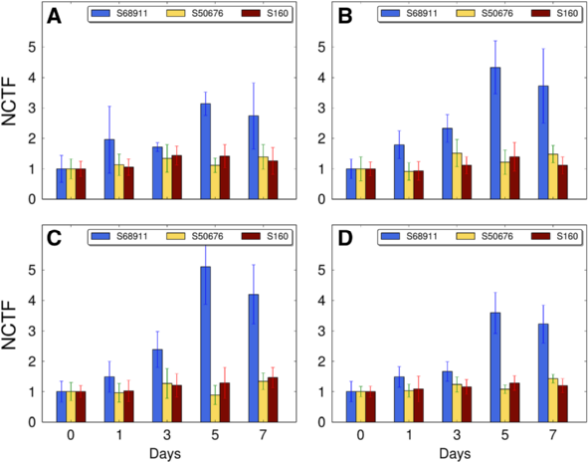
Changes in cell wall cellulose content in various cell types upon cold treatment. Plants of three maize inbred lines were grown at 24 °C/22 °C (control, day 0) and transferred to 14 °C/12 °C for seven days. Third leaves were collected at time points indicated and cross-sections were stained for cellulose with Calcofluor White. Fluorescence was quantitated under a confocal microscope and expressed as normalized corrected total fluorescence (NCTF) as described in Materials and Methods. All values are expressed relative to the mean fluorescence at day 0 for a given line and cell type set at 1. **a** Vascular tissue; **b**. Bundle sheath; **c**. Kranz mesophyll; **d**. Whole veins. Data are means ± SD for at least 10 veins from three plants from three independent experiments

The cell wall properties were characterized by measuring the activity of cell-wall-associated pectin methylesterase (PME) and peroxidases (POX) (Fig. 7), and by visual estimation of the cellulose content following staining with Calcofluor White (Fig. 8). The both enzyme activities showed a transient increase in all three inbred lines after 14 h (night + 4 h of day) of chilling, the strongest in the S68911 line, particularly in the case of POX. In contrast, the Calcofluor White signal increased substantially only in S68911 and was virtually constant or showed only minor changes in the two other lines.

**Fig. 9:**
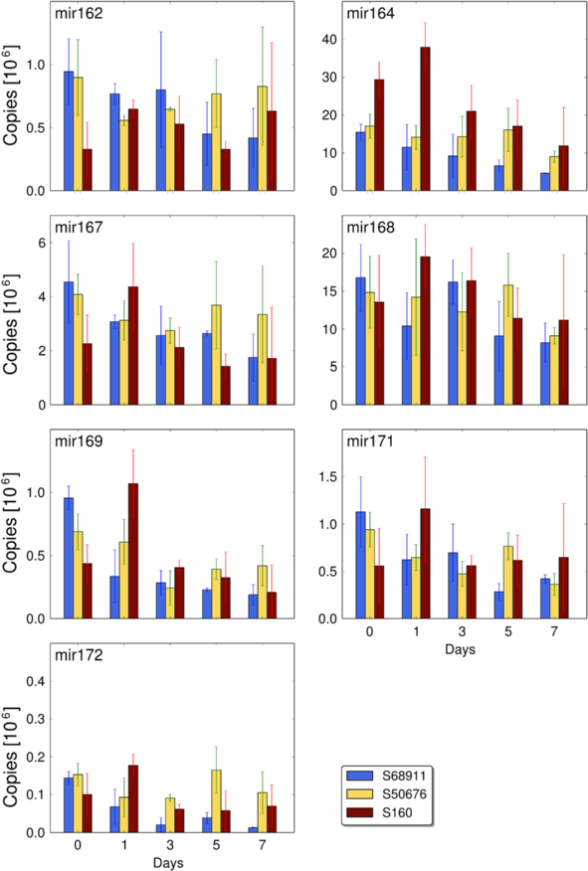
Changes in miRNA levels upon cold treatment. Plants of three maize inbred lines were grown at 24 °C/22 °C (control, day 0) and transferred to 14 °C/12 °C for seven days. Third leaves were collected at time points indicated, total RNA was isolated and individual miRNA species were quantitated as described in Materials and Methods. Data are means ± SD for three independent experiments with three plants each

The fact that the three lines studied here show developmental differences upon cold treatment was apparent from the field (Fig. 1) and growth-chamber (Fig. 2) observations. They were in line with the transcriptomic data and indicated that the cold-tolerance of the S68911 line could be based on the induction of developmental and/or acclimatory processes under adverse conditions, in contrast to the S50676, and particularly the most cold-sensitive S160, inbred lines generally showing gene repression. Since only the S68911 line showed a strong repression of the *DCL1* gene whose product is engaged in miRNA maturation, and miRNAs often act as negative regulators of developmental processes, we decided to look directly for cold-induced changes in miRNA levels. We chose seven miRNA species, homologs of *Arabidopsis* DCL1-dependent miRNAs of known regulatory roles. Figure 9 shows how the abundance of these miRNAs changes during a week-long moderate-cold treatment in all three maize lines. The miRNAs studied were expressed at strikingly different levels, from very low (miR172) to very high (miR164, miR168). Notably, despite those differences, all seven miRNAs showed a highly consistent pattern of changes: a progressive depletion in S68911 and a marked but transient increase followed by a slow return to the control level in the S160 line. S50676 showed a pattern intermediate between those two: a fairly constant level throughout the week of cold treatment or an initial slight depletion followed by a return to the basal level. Thus, the observed patterns of miRNA expression confirm the original assumption regarding different developmental responses of the studied lines to the cold.

## Discussion

Maize is generally considered a cold-sensitive species, although some authors underline its strong diversity in this respect [31], likely due to the extraordinary genetic variability. Based on this feature, artificial selection has allowed recent and ongoing remarkable progress in the maize spread throughout different climates, including temperate ones with a short vegetative period limited by low temperatures. Some modern maize inbred lines perform well in the field under severe cold and even can survive short-term frost spells. These materials, here represented by the S68911 inbred line, seem to be a good model for studying the maize response to low temperatures with the goal of discovering the mechanism of the attained cold-tolerance.

The S68911 line not only showed a perfect early vigor under cold spring conditions, but also outperformed the two other lines studied here at moderately low temperatures under controlled conditions. Moreover, only this inbred line had the ability to improve the efficiency of the photosynthetic apparatus at severe cold following a moderately-low temperature pretreatment, *i.e.*, exhibited symptoms of acclimation [10]. Also earlier studies on diverse maize inbreds have demonstrated that chilling spells of several days may improve the maize cold-tolerance [8, 11–15]. However, the molecular mechanisms of that effect have not been studied comprehensively until present.

The transcriptome profiling of three inbred lines performed here to search for the molecular mechanism of the maize response to moderately low temperatures showed thousands of genes responding to chilling. Many more such genes were found for the S68911 and S50676 lines than for S160. The S160 line also demonstrated the weakest expression change of specific genes (those affected in that line only). S68911 and S50676 belong to the Stiff Stalk Synthetic (SSS) pool whereas S160 to the 354 pool. The former pool gave rise to numerous inbred lines produced in both national and private sectors [32], including the B73 line whose genome – as the first sequenced genome of maize [2] - is the reference for the community of maize scientists. The 354 gene pool is markedly distant from SSS [29], therefore one may suppose that the observed diverse transcriptional response to the cold could at least in part reflect the different genetic backgrounds of the lines.

The differences in the number of responding genes between the lines from the two gene pools hinders interpretation of the results of global analysis among the inbreds, since the over-representation of a given GO class depends to some extent on the overall number of responding genes. Despite that difficulty, a response to low temperature common to the three lines comprising repression of large groups of genes related to photosystems (GO:0009521, Cellular Component class) and cell redox homeostasis (GO:0045454, Biological Process class) can be recognized easily. This common response confirms the widely held view that the photosynthetic apparatus and redox balance are major targets of low temperature in maize [33]. On the other hand, the global analysis indicated up-regulation of numerous genes related to the mitochondrion (GO:0005739, Cellular Component class). The literature on the role of mitochondria in cold-treated maize is scarce [12, 34], therefore this finding is noteworthy. Another interesting result of the global analysis is the over-representation of induced genes related to DNA conformation changes (GO:0071103, Biological Process class), likely underlying and/or reflecting the massive changes in gene expression upon cold treatment found in this study, and to signal transduction (GO:0032561, Molecular Function class), indicative of metabolic changes.

High-throughput methods are focused on global analyses and therefore do not allow a straightforward selection of genes of particular biological relevance. To compensate for this inherent deficit we attempted to identify individual genes responsible for the different cold-tolerance levels of the inbred lines studied. To this end, we used two approaches. First, by means of clustering, we selected genes showing up-regulation or down-regulation in a single inbred line only. Such genes (called by us “specific”) were then analyzed for their functions to select those of potential importance in cold acclimation. The second, independent approach involved a filtering protocol for genes showing expression in control conditions and a significant expression change in cold-treated plants in one inbred line only (“unique” genes). Both approaches highlighted the exceptionality of the S68911 line. First, only that line demonstrated a clear-cut dominance of induction over repression of specific genes, in contrast to the two other lines where repression was predominant. Interestingly, a similar phenomenon, *i.e.*, the prevalence of up-regulation over down-regulation in response to cold treatment was found in a cold-tolerant genotype of rice, another thermophilic grass crop [35]. Second, all four unique genes identified were expressed in the S68911 line. One may thus speculate that the exceptional cold-tolerance of the S68911 line is founded in its specific transcriptional response and, consequently, a thorough analysis of the S68911-specific and unique genes should greatly enhance our understanding of the maize adaptation to the temperate climate.

The exceptional character of the S68911 line is also well pronounced when one considers defined groups of cold-responsive genes rather than their whole pool. Using the simple criterion of (predicted or experimentally verified) subcellular localization of proteins one can infer the biological role of the genes of interest. Again, for virtually all such localizations the subsets of line-specific cold-affected genes show clear-cut dominance of induction over repression in S68911 only. Three such subsets stand out both with respect to the overwhelming predominance of induction and the importance of their products. The first comprises genes encoding chloroplast proteins. This finding corresponds remarkably with the line-specific ability of S68911 to acclimate the photosynthetic apparatus to severe cold at a moderately low temperature found earlier in the present study. One may thus conclude that the general cold-susceptibility of the photosynthetic apparatus documented here as an enrichment of GO category “Photosystem” among down-regulated genes in all tested lines could be overcome in S68911 by the line-specific up-regulation of other chloroplast-related genes.

The two other groups of genes up-regulated specifically in the S68911 line are those encoding proteins related to the nucleus, including many transcription factors, and those likely involved in cell communication with the plant’s internal milieu (encoding proteins of the cell wall, plasmalemma, plasmodesmata, and Golgi apparatus). On that basis it may be speculated that the specific mechanisms responsible for the cold-tolerance of the S68911 line could involve modification of developmental processes. The changes in the expression of numerous genes related to cell wall modification and cell-to-cell transport could reflect an increased demand for extracellular signaling and/or metabolite trafficking. Interestingly, among the latter genes are several related to plasmodesmata. These structures have earlier been shown to regulate the metabolite transport in maize leaves at low temperature: they closed in a chilling-sensitive line and remained open in a chilling-tolerant one [36]. The mechanism of the phenomenon is not clear, but recent data shows that signal transduction between the nucleus, plastids and plasmodesmata mediated by redox potential regulates the latter [37].

One should note that the four unique genes expressed solely in the S68911 line also represent the processes and cellular localizations predicted to be important for the maize cold tolerance: three are related to the protoplast exterior (two genes are connected with cell wall biogenesis and one could be involved in cell communication with the plant’s internal milieu), and one encodes a transcription factor. Remarkably, one of these genes, GRMZM2G331566, encoding a hypothetical cellulase (http://maizecyc.maizegdb.org/MAIZE/NEW-IMAGE?type=GENE&object=GRMZM2G331566), shows a major nucleotide sequence divergence of its regulatory region between S68911 and the two other lines studied. In the reference B73 inbred line this gene is expressed solely in generative organs, cobs and tassels (http://maizegdb.org/gene_center/gene?id=GRMZM2G331566), and we did not find its transcript in leaves of several other maize lines studied earlier, either [23, 24]. This vegetative-organ expression unique to the S68911 line could be due to the specific nucleotide sequence of the gene’s 5’ flank. Potential recognition sites for two transcription factors, MYB15 and MYB84, are present only in the S68911 variant of the gene. It is of utmost interest that the latter itself is cold-induced at the transcript level in another maize line studied by us (Jończyk et al., manuscript in preparation); moreover, MYB84 binding sequences are abundant in maize cold-induced genes (*ibid*.). It should be noted, however, that for the three other unique genes we found no nucleotide sequence divergence between S68911 and the other lines, so the reason for their unique expression in the S68911 line must lie elsewhere and likely involves factors acting *in trans*. For clarification we stress here that the “uniqueness” of all four genes in question has been confirmed by RT-qPCR, which means that their contrasting levels of expression between S68911 and the other lines are real and are not simply due to poor hybridization to microarray probes as a consequence of the divergence of the nucleotide sequences of their mRNAs.

Transcriptomic data are a useful indirect indication of processes affected but do not constitute an unequivocal proof of the nature of the physiological response. To address directly the physiological processes suggested by the transcriptomic data as potentially responsible for the differences in the cold-sensitivity of the lines studied we examined some aspects related to cell wall modifications and to the regulation of development. We chose activities of cell-wall-associated enzymes known to be engaged in stress responses in other plants, POX and PME, the latter shown to be involved in frost-hardiness of rape [38]. Changes in the activities of these two enzymes in response to the cold were observed in all three lines, the strongest in S68911. Thus, the difference between the lines was quantitative rather than qualitative as was expected from transcriptomic data analysis. The changes were fairly rapid and transient, representing therefore the alarm phase of stress [10]. Such changes are often involved in stress signaling [39]. The role of the cell wall in the signal transduction to protoplasts [40], and of cell-wall peroxidases in stress response [41] is known. Thus, the present enzymatic study confirms our earlier hypothesis based on transcriptomic data for maize treated with severe cold stress [24] that the cell wall itself and/or the cell wall/membrane interactions could act as a receptor of the low temperature stress in maize.

One of the effects of the induction of signal transduction during the alarm phase of stress, as manifested by the changes in PME and POX activities, could be modification of cell walls. This was indeed observed by microscopic examination of cell walls in maize leaf preparations labeled with Calcofluor White which binds to cellulose [42, 43]. The increased cellulose content of the cell walls, again most pronounced in the cold-tolerant S68911 line, seems to be long-lasting, so it could reflect the acclimation phase of stress [10]. The role of cell wall modifications in the maize acclimation to cold is not clear. One may posit that it protects cells against water leakage caused by severe cold: dehydration related to diverse stresses can be limited by rigidification of cell walls [41]. Secondary water stress induced by cold treatment and improvement of the water potential by acclimation at a moderately low temperature in cold-tolerant maize inbred lines have been reported [8], but not in the context of cell wall properties. Thus, this phenomenon deserves further studies.

Another effect of the cold apparent at the transcriptome level was the modification of developmental processes. Their nature is impossible to deduce on the basis of the transcriptome changes alone and should be determined using direct approaches, albeit we suggest a likely direction. The experiments were performed on a fully developed third leaf whose maturation had already been completed. One should note, however, that this leaf – the first fully autotrophic one in a young seedling [44] – has a life-span of a dozen days only [28], concluding with senescence, which can be accelerated by low temperature [45]. Arresting this process by cold-acclimation would clearly improve the leaf performance at severe cold and during the recovery period. Notably, we found that seedlings of the S68911 line did not show any symptoms of injury during recovery from a four-day severe cold stress applied after four days of acclimation at a moderately low temperature. This tolerance contrasted with the behavior of the two other lines tested, where symptoms of injury and senescence did develop during recovery.

Since the transcriptomic data were not specific enough to suggest a particular aspect of development as the most relevant, and the physiological response of the S68911 line to the cold had not been studied before, we decided to address this question in a broad manner and looked for changes in the pattern of miRNAs, the known regulators of numerous developmental events [46]. We chose for quantitation several miRNA species known to be processed by DCL1, whose gene *DCL1* was found to be strongly repressed exclusively in the S68911 line. In agreement with that repression, all seven miRNAs assayed showed a pronounced drop in abundance in cold-treated S68911. Since miRNAs generally inhibit developmental processes, their down-regulation indicates induction of such processes, in agreement with the transcriptomic data. Regarding the miRNAs, the S50676 line, as expected, showed no marked changes on cold treatment, while S160 behaved in an unexpected manner by showing their transient up-regulation. That increased abundance of the miRNAs was unlikely to be caused by DCL1 up-regulation since we found no change in *DCL1* expression in cold-treated S160 line. Whatever the mechanism of that increase, it signals repression of developmental processes in the most cold-sensitive line studied here.

Among the miRNAs tested, miR172 seems of particular interest since it supports the transition of the leaf from juvenile to adult phase in maize by down-regulating a negative regulator of that transition [47]. Thus, the decrease of miR172 level found in our study in S68911 would suggest an arrest of this transition by the cold. A note of caution is in order here, since in our study miR172 was quantitated in the middle part of a fully developed leaf while [ibid.] investigated the apex and the basal (youngest) part of developing leaves, so a direct comparison of the results of these two studies is not justified.

All seven miRNAs tested here and found to be depleted by cold treatment in the cold-tolerant S68911 line have earlier been shown in other plant species to be affected by diverse abiotic stresses: drought, low nitrate availability, and salinity [48–54]. Basing on the present data, we add cold stress to that list, with the limitation that it is inbred-line-specific. A similar line-specific miRNA response to abiotic stresses has already been shown in maize [55].

Using the terms of classical physiology of stress [10], maize has adapted to the temperate climate by shortening its life cycle, *i.e.*, by the avoidance mechanism. This was achieved by breeding for early flowering, which enabled the life cycle to be completed during the relatively short vegetative season with temperatures higher than *ca.* 8 °C. Further adaptation could be obtained by lowering this temperature limit, particularly for the early growth stages, since it would prolong the period of vegetative growth. It seems that breeders are close to achieving this goal, as exemplified by the S68911 inbred line showing an exceptional ability to survive periods with temperatures below that limit under field conditions. This trait shows that maize is intrinsically able to be adapted to the cold, possibly owing to its exceptional genetic plasticity.

## Conclusions

By combining physiological and molecular approaches we have identified three mechanisms likely responsible for the cold-tolerance of the S68911 line. One is related to acclimation of the photosynthetic apparatus, the second to the cell wall structure, which seems to be modified already at a moderately low temperature, thereby facilitating withstanding the cold stress, and the third involves modifications of developmental processes. The major role of these mechanisms in the cold-tolerance of the S68911 line was deduced from the changes at the transcriptome level and found good support in functional studies. However, more biochemical, molecular and physiological studies on S68911 and other cold-tolerant lines are needed to provide full understanding of the mechanisms of their departure from the original cold-sensitivity of maize. Similarly as in the case of the domestication of teosinte, the wild ancestor of maize [56], one cannot exclude that changes at the level of single genes were crucial for the development of cold-tolerance in maize. Indeed, the early flowering trait mentioned earlier, allowing adaptation to the temperate climate is likely due to a deletion of a single gene, *Dwarf8*, found at a very high frequency in Northern Flint genotypes and almost absent in tropical ones [57]. In the present study we found four genes expressed uniquely in the S68911 line, one of which shows a substantial divergence of its upstream (regulatory) region. This gene codes for a putative cellulase, which indicates its likely involvement in cell wall modification, in line with other conclusions of this work. How the cold-repressible action of the encoded cellulase could promote the cold-tolerance of the S68911 line remains to be established. Similarly, the exact mechanisms by which a general de-repression of developmental processes indicated by miRNA down-regulation upon cold treatment leads to improved cold-tolerance also need to be solved. The presented results do not provide a definite answer to these questions, but allow focusing further studies on a few selected and promising aspects.

## Methods

### Plant material

Three inbred lines of dent type maize (*Zea mays* spp. *indentata*) were selected for the project on the basis of field observations: S68911 and S50676 (pedigree Stiff Stalk Synthetic/Iodent), and S160 (pedigree 354), produced by Plant Breeding Smolice Ltd., Co., Poland. The selection data derived from routine observations at the Smolice (West Poland) location in years 2004, 2006 and 2007. At this location maize is sown routinely in the second half of April, when the soil temperature is higher than 8 °C. The average temperature of May is between 10 and 15 °C (Fig. 1). Seedling performance was characterized by early vigor evaluated in the week directly following the cold spell common (long-term probability of occurrence of 33 %) in the Central European climate in mid-May, and the effective temperature sum (ETS). Early vigor was estimated visually in a 1-9 scale at the stage of 4th leaf, assuming 1 as the weakest early vigor (96 – 100 % injured or necrotic plants), 9 as the highest one (0 – 5 % injured or necrotic plants), with successive steps differing by ten percentage points [58]. The ETS from sowing to 50 % of silking was calculated as described in [59], taking 6 °C as the base temperature.

### Growth under controlled conditions

Kernels were germinated in wet sand for three days in darkness at 25 °C. Then seedlings were transferred to pots containing Knop’s solution and were grown in a growth chamber (photoperiod 14 h/10 h day/night, light irradiance 250 μmol quanta m^-2^ s^-1^, temperature 24 °C/22 °C and relative humidity 60 %/80 %) until the third leaf (V3 stage) was fully developed (*i.e*., the ligular region of the leaf was formed). Then the plants were taken for experiments (control) or were subjected to various low-temperature regimens as specified below. Four fully independent consecutive biological experiments were performed for microarray and RT-qPCR experiments and three for other purposes.

### Testing for cold-acclimation at moderately low temperature

Plants at the V3 stage were transferred to 14 °C/12 °C (moderately low temperature) for four days, then to 8 °C/6 °C (severe cold stress) for four days and finally to 24 °C/22 °C for two days (regrowth). At the end of each period the plants were photographed.

The maximum quantum efficiency of PSII primary photochemistry (Fv/Fm) and PSII operating quantum efficiency (Φ_PSII_) were measured with a fluorometer (PAM 200, H. Walz, Germany) at time points specified in the legend to Fig. 3. Separate measurements were done for plants transferred directly (without a cold-acclimation period) from 24 °C/22 °C to 8 °C/6 °C. For the measurement of Fv/Fm, the saturating one-second light flash intensity was about 3500 μmol quanta m^-2^ s^-1^. Before the measurements the plants were dark-adapted for 30 min at 24 °C. For the measurement of Φ_PSII_, leaves were exposed to eight two-minute periods of light of 200 μmol quanta m^-2^ s^-1^ intensity, each period terminating with a saturating one-second light flash of *ca.* 3500 μmol quanta m^-2^ s^-1^. Independent experiments were repeated three times with three to five plants per line per experimental variant.

### Plant sampling for microarray and RT-qPCR experiments

At the end of the light period half of the plants were transferred to 14 °C/12 °C (day/night) without changing the other conditions; the other half were grown in the same conditions as before (control plants). After a 38-hour cold-treatment (dark period + light period + dark period + 4 h of light period), the middle part of the third leaf was collected from three plants for each treatment and inbred line, pooled, flash-frozen in liquid nitrogen and stored at -80 °C until RNA isolation.

### Microarray description

Microarrays designed and produced by the Maize Oligonucleotide Array Project, University of Arizona, Tucson, AZ, USA [60] were used. A single slide comprised 46,128 probes, mainly 70-mer, but also some 40- and 50-mer, as well as positive, negative and print controls.

### RNA isolation, amplification and hybridization

RNA isolation, purification, amplification, labeling and hybridization were performed according to the procedure of the microarray producer, with some modifications. Briefly, RNA was isolated and purified from frozen leaf samples with RNeasy Plant Mini Kit (Qiagen) following manufacturer’s manual, checked for quality by gel electrophoresis, and quantified with an ND-1000 spectrophotometer (Nanodrop). cDNA was synthesized with Message Amp-II Kit (Ambion) and cleaned with DNA Clear Kit (Ambion) according to manufacturer’s instructions. The cDNA was transcribed with T7 RNA polymerase for 18 h at 37 °C in a mixture of ATP, CTP, GTP, UTP and aaUTP (3:3:3:1:2), in 10 x reaction buffer. The obtained aRNA was cleaned with MEGA Clear Kit (Ambion), vacuum-dried and dissolved in 0.2 M NaHCO_3_. Samples were labeled with Cy3 or Cy5 monoreactive dye (Amersham Pharmacia) in darkness at room temperature. The reaction was terminated with 4 M hydroxylamine and unincorporated dye was removed using Nucleospin RNA Clean-up column (Macherey-Nagel). Before hybridization, DNA probes on microarray slides were immobilized by re-hydration and UV cross-linking [60]. The slides were pre-hybridized in 5 × Saline Sodium Citrate (SSC), 0.1 % Sodium Dodecyl Sulphate (SDS), 1 % Bovine Serum Albumin (BSA) (Sigma-Aldrich) for 45 min at 42 °C, washed twice with distilled water for 5 min, once with 100 % EtOH for 2 min and spin-dried. Hybridization with the Cy3- and Cy5-labeled aRNA was performed in buffer containing 50 % formamide, 5 × SSC, 0.1 % SDS, 10 μg ml^-1^ yeast tRNA, 10 μg μl^-1^ salmon sperm DNA in AHCXD Extra Deep Hybridization Cassettes (ArrayIt) for 16 h at 42 °C. Then the slides were washed at 42 °C in 2 × SSC, 0.1 % SDS for 5 min, 0.1 × SSC for 5 min, and 0.05 × SSC for 10 min, and spin-dried. Immediately after the washing the microarrays were scanned with GenePix 4000B (Molecular Devices) and feature extraction was performed with GenePix Pro 3.0 software.

### Data normalization and statistical analysis

The hybridizations were performed in a loop design with cross hybridizations [61]. Separate sets of hybridizations were run for each of the four biological replications. To minimize the effect of potential dye-bias, the Cy3 and Cy5 dyes were swapped for half of the replications. A total of 48 hybridizations were performed.

The raw microarray scanning data were first complemented with missing data by the Bayesian Principal Component Analysis (BPCA) method [62] with the use of JBPCAFill software (http://ishiilab.jp/member/oba/tools/JBPCAFill.html). Then the data were normalized across slides by the print-tip loess method (Acuity 4.0, Molecular Devices) and between hybridizations by the loess method (JMP Genomics 6.03, SAS Institute). Data were analyzed statistically (ANOVA) with the JMP Genomics 6.03 software. The obtained p-values were then corrected for multiple comparisons with the False Discovery Rate [63] set at 0.01 or 0.05.

The microarray experiments were described in compliance with the MIAME (Minimum Information About Microarray Experiment; [64] guidelines. The raw and normalized data have been deposited at the ArrayExpress database (www.ebi.ac.uk/arrayexpress) under experiment accession number E-MEXP-3508.

### Data analysis

Clustering was done with JMP Genomics 6.03 (SAS Institute). The number of clusters was set arbitrarily at 30. Global analysis was performed with the Gene Ontology hierarchical system and enrichment analysis. GO annotations were assigned to probes as described before [65]. Over-represented GO categories were detected with the Ontologizer program [66] using the “Parent-Child Union” method which takes into account the hierarchical structure of the GO system, and the FDR correction [63] set at 0.05. When several probes matched a single gene, it was counted only once, and genes without a GO annotation were excluded from analysis. The GO graphs were drawn with the GraphViz software (www.graphviz.org). Detailed analysis was based on literature data mining with Pathway Studio 9.0 (Elsevier). To use this program the identifiers of the genes of interest were converted to entrez ids accepted by the software, as described earlier [65]. Additional annotations were retrieved from the Gramene website (http://ensembl.gramene.org/Zea_mays/ Info/ Index) and the InterPro database (http://www.ebi.ac.uk/interpro).

### Sequencing of putative promoter regions of unique genes

DNA was isolated and purified from leaves of all three maize lines using DNeasy Plant Maxi kit (Qiagen) and required fragments were PCR-amplified using Phusion Hot Start II High-Fidelity polymerase (Fermentas) and 35 cycles of (98 °C 10 s, 61 °C 30 s, 72 °C 30 s) preceded by initial denaturation at 98 °C for 30 s and followed by final extension at 72 °C for 10 min. For the GRMZM2G331566 gene corresponding to the MZ00026395 probe the primers used were CCCTGAGCAACGAAAGAGC and TTACCTTGGCGGAGTGACC amplifying a 1008-bp fragment from position -770 to +238, where position +1 is A in the AUG start codon (numbering according to the reference B73 sequence). Sequences of primers for the other genes are available on request. Amplification products were cloned in pJET1.2/blunt using CloneJET PCR Cloning kit (Fermentas) and sequenced using Sanger chemistry and universal primers in a commercial facility. At least three independent clones sequenced at both strands were taken for each gene and maize line. The obtained sequences were compared with one another and with the reference B73 line using ApE v 2.0.45 (http://biologylabs.utah.edu/jorgensen/wayned/ape/). Potential transcription-factor-binding sequences were identified using the JASPAR database (http://jaspar.genereg.net) and TFBSTools package [67] for the R software [68].

### Quantitative real-time PCR

Quantitative real-time PCR primers were designed for 29 transcripts representing different levels of expression. As reference, glyceraldehyde 3-phosphate dehydrogenase gene (*GAPDH*) transcript was used because it showed constant expression in all experimental variants. The primers (Additional file 3) were designed using the open access software from PREMIER Biosoft International (NetPrimer: www.premierbiosoft.com/ netprimer) and Invitrogen. One of the primers from each pair was designed to hybridize to a sequence corresponding to a fragment of the microarray probe.

The plant material for RT-qPCR analyses was derived from the same four biological experiments as for microarray analyses, but RNA was isolated from separate plants. The entire procedure of cDNA synthesis and RT-qPCR has been described elsewhere [24] and quality of the results was verified routinely by performing melting curves for the PCR products and analyzing their purity by agarose electrophoresis. Real-time PCR was carried out in a MyiQ2 (Bio-Rad) thermocycler. The data were analyzed with iQ5 Optical System Software (Bio-Rad). The Ct value was determined by the iQ5 software for all the samples and mean values were used in the formula:

$$ \mathsf{\varDelta \varDelta Ct} = \left[\left(\mathsf{C}\mathsf{t}\ \mathsf{G}\mathsf{O}\mathsf{I}\ \mathsf{K}\ \hbox{-}\ \mathsf{C}\mathsf{t}\ \mathsf{H}\mathsf{K}\mathsf{G}\ \mathsf{K}\right)\ \hbox{-}\ \left(\mathsf{C}\mathsf{t}\ \mathsf{G}\mathsf{O}\mathsf{I}\ \mathsf{C}\ \hbox{-}\ \mathsf{C}\mathsf{t}\ \mathsf{H}\mathsf{K}\mathsf{G}\ \mathsf{C}\right)\right], $$

where Ct GOI and Ct HKG are the threshold cycles of the gene of interest and the house-keeping reference gene (here, *GAPDH*) [69], respectively (K – control sample, C – sample obtained from cold-treated plants). To confirm the linearity of the assay, amplification efficiencies of the target and reference genes were determined from a dilution series of sample cDNA and were found to be close to 100 % for all genes.

### Cell wall properties

Plants grown at 24 °C/22 °C as described were transferred to 14 °C/12 °C at the end of the light period and grown for a further seven days. Leaves were harvested at the last day of growth at the optimal temperature (control) and at the end of the first, third, fifth, and seventh day of chilling. Cell walls from leaf laminas were prepared using a modified method of [70]. Fresh leaf tissue was homogenized in a Waring blender in 0.05 M HEPES buffer, pH 6.8, containing a mixture of protease inhibitors (PMSF, aprotinin, bestatin, pepstatin A and leupeptin, 1 mM each), filtered through miracloth and rinsed several times with cold water. After air drying, dry mass of crude cell wall preparations was determined by weighing.

Cell wall proteins were extracted from fresh crude cell wall preparations with 0.05 M HEPES buffer, pH 6.8, containing 1 M NaCl and the mixture of protease inhibitors under gentle agitation at 4 °C for 2 h followed by centrifugation (10 min, 12,000 x *g*). The protein extracts were used for determination of pectin methylesterase (PME) and peroxidase (POX) activities.

PME activity was determined according to [71]. Reaction mixture contained 0.5 % (w/w) highly-methylated citrus pectins (Sigma, Germany), 0.2 M NaCl and 0.015 % (w/v) Methyl Red (Sigma, Germany) as a pH indicator. Cell-wall extract sample (100 μl) was added to 900 μl of the reaction mixture in a microcuvette. Color changes from yellow to red (due to pH lowering upon pectin de-esterification) were measured spectrophotometrically at 525 nm (Shimadzu, Japan) for 3 min at 25 °C. A calibration curve was obtained by adding 1 to 200 nEq of H^+^ to 1 ml of reaction mixture. The enzyme activity was expressed in arbitrary units (one unit is the amount of enzyme releasing one nEq of H^+^ in one minute) per gram of cell wall dry matter. Peroxidase activity was analyzed at 25 °C according to [72] and [73] in 0.05 M Na-acetate buffer, pH 5.0, containing 2 mM guaiacol and 100 μl of cell wall extract. The reaction was initiated by the addition of 10 μl of 30 % H_2_O_2_ and absorbance was recorded at 436 nm for 2 min (linear phase). The enzyme activity was expressed in arbitrary units (one unit is the amount of enzyme increasing the absorbance by 0.1 in one minute per gram of cell wall dry matter).

For microscopic observations, third-leaf fragments from plants grown as described above were fixed in 2 % (w/v) paraformaldehyde and 2 % (v/v) glutaraldehyde, dehydrated in a graded ethanol series and embedded in LR White. Thin sections (20 μm) were mounted on glass slides and stained with 1 mg/ml Calcofluor White (Sigma Aldrich) for 10 min. After rinsing, the sections were viewed in a Nikon A1R MP confocal microscope (excitation/emission, 405/488 nm), and small and medium-size veins were observed. To allow comparison of results within a given maize line, scan conditions optimized for the sections from plants cold-treated for seven days, where the highest fluorescence was observed, were used for all samples. Fluorescence was analyzed using ImageJ software (www.imagej.net). Corrected Total Fluorescence (CTF) was determined separately for vascular elements (VE), bundle sheath cells (BS), Kranz mesophyll cells (KMS), and for whole veins using the following equation:

$$ \mathsf{C}\mathsf{T}\mathsf{F} = \mathsf{I}\mathsf{D}\_\mathsf{R}\mathsf{O}\mathsf{I}\ \hbox{-}\ \mathsf{A}\_\mathsf{R}\mathsf{O}\mathsf{I} \times \mathsf{B}\mathsf{M}, $$

where ID_ROI is integrated density of the field of interest, A_ROI its area, and BM is background mean grey value. For clarity, for each compartment (VE, BS, KMS, veins) and each inbred line CTF values were normalized against relevant mean CTF values from control plants. For each experimental variant, 7-15 veins from three different plants were analyzed.

### miRNA quantitation

Nucleotide sequences of maize miRNAs orthologous to those known to require DCL1 for maturation in *Arabidopsis* were taken from the miRNA database at http://www.mirbase.org/. The best matches indicating likely true homology were obtained for seven miRNA species: miR162, 164, 167, 168, 169, 171, and 172.

RNA was isolated from the third leaves of plants grown under control conditions or subjected to moderate chilling, as described above, for 1, 3, 5, and 7 days, and individual miRNAs were quantitated by RT-qPCR according to [74]. Primers for miRNA quantitation were either those reported earlier by [75] (for zma-MIR164a/b/c/d/g, zma-MIR167a/b/c/d, zma-MIR168a/b, and zma-MIR172b/c/d) or were designed by us using the rules specified by those authors (for zma-MIR162a, 169a/b, and 171a/d/e/i/j/n; primer sequences are given in Additional file 26). All primers were obtained from Sigma. Chemicals for reverse transcription were obtained from Thermo Scientific. For all primer pairs standard curves (seven consecutive tenfold dilutions, from 0.1 ng/μl, nine for zma-MIR172) were obtained using synthetic miRNAs obtained from Sigma. The size of PCR products obtained on maize RNA preparations was checked by electrophoresis in 5 % agarose, and additionally for zma-MIR164 and zma-MIR172 the products were cloned and sequenced. miRNA abundance was calculated per microgram of total RNA preparation using three independent biological replications, each determined in triplicate for each data point. RT-qPCR reactions for each experimental variant and biological replication were run in triplicate (technical replication).

## Additional files

Additional file 1:
**Rough data for field parameters of selected maize inbred lines at Smolice location in years 2004, 2006, and 2007.** (PDF 24 kb) Additional file 2:
**Temperatures at Smolice location in 2004, 2006, and 2007.** (PDF 241 kb) Additional file 3:
**Primers for RT-qPCR.** (PDF 219 kb) Additional file 4:
**RT-qPCR versus microarray results.** (PDF 185 kb) Additional file 5:
**Gene Ontology categories of GO class “Molecular Function” significantly over-represented among transcripts up-regulated by cold treatment in S68911 inbred line.** (PDF 227 kb) Additional file 6:
**Gene Ontology categories of GO class “Molecular Function” significantly over-represented among transcripts down-regulated by cold treatment in S68911 inbred line.** (PDF 157 kb) Additional file 7:
**Gene Ontology categories of GO class “Cellular Component” significantly over-represented among transcripts up-regulated by cold treatment in S68911 inbred line.** (PDF 181 kb) Additional file 8:
**Gene Ontology categories of GO class “Cellular Component” significantly over-represented among transcripts down-regulated by cold treatment in S68911 inbred line.** (PDF 159 kb) Additional file 9:
**Gene Ontology categories of GO class “Biological Process” significantly over-represented among transcripts up-regulated by cold treatment in S68911 inbred line.** (PDF 247 kb) Additional file 10:
**Gene Ontology categories of GO class “Biological Process” significantly over-represented among transcripts down-regulated by cold treatment in S68911 inbred line.** (PDF 223 kb) Additional file 11:
**Gene Ontology categories of GO class “Molecular Function” significantly over-represented among transcripts up-regulated by cold treatment in S50676 inbred line.** (PDF 235 kb) Additional file 12:
**Gene Ontology categories of GO class “Molecular Function” significantly over-represented among transcripts down-regulated by cold treatment in S50676 inbred line.** (PDF 233 kb) Additional file 13:
**Gene Ontology categories of GO class “Cellular Component” significantly over-represented among transcripts up-regulated by cold treatment in S50676 inbred line.** (PDF 226 kb) Additional file 14:
**Gene Ontology categories of GO class “Cellular Component” significantly over-represented among transcripts down-regulated by cold treatment in S50676 inbred line.** (PDF 224 kb) Additional file 15:
**Gene Ontology categories of GO class “Biological Process” significantly over-represented among transcripts up-regulated by cold treatment in S50676 inbred line.** (PDF 246 kb) Additional file 16:
**Gene Ontology categories of GO class “Biological Process” significantly over-represented among transcripts down-regulated by cold treatment in S50676 inbred line.** (PDF 253 kb) Additional file 17:
**Gene Ontology categories of GO class “Molecular Function” significantly over-represented among transcripts up-regulated by cold treatment in S160 inbred line.** (PDF 174 kb) Additional file 18:
**Gene Ontology categories of GO class “Cellular Component” significantly over-represented among transcripts up-regulated by cold treatment in S160 inbred line.** (PDF 244 kb) Additional file 19:
**Gene Ontology categories of GO class “Cellular Component” significantly over-represented among transcripts down-regulated by cold treatment in S160 inbred line.** (PDF 155 kb) Additional file 20:
**Gene Ontology categories of GO class “Biological Process” significantly over-represented among transcripts up-regulated by cold treatment in S160 inbred line.** (PDF 255 kb) Additional file 21:
**Gene Ontology categories of GO class “Biological Process” significantly over-represented among transcripts down-regulated by cold treatment in S160 inbred line.** (PDF 253 kb) Additional file 22:
**Possible functions and cellular localization of products of genes specific to S68911 inbred line.** (XLSX 86 kb) Additional file 23:
**Possible functions and cellular localization of products of genes specific to S50676 inbred line.** (XLSX 92 kb) Additional file 24:
**Possible functions and cellular localization of products of genes specific to S160 inbred line.** (XLSX 168 kb) Additional file 25:
**Comparison of nucleotide sequences of putative promoter region of GRMZM2G331566 gene.** (PDF 246 kb) Additional file 26:
**Primers for miRNA.** (PDF 8 kb)
