# Supplementary material for: Molecular foundations of chilling-tolerance of modern maize
Source: BMC Genomics. 2016 Feb 20;17:125. doi: 10.1186/s12864-016-2453-4 (PMC4761173; doi:10.1186/s12864-016-2453-4)
Supplement: Additional file 1: — Rough data for field parameters of selected maize inbred lines at Smolice location in years 2004, 2006, and 2007. (PDF 24 kb) [file 12864_2016_2453_MOESM1_ESM.pdf]

Additional file 1. Rough data for field parameters of selected maize inbred lines at Smolice location in years 2004, 2006, and 2007.

Inbred lines	sowing date	silking			pollen shed			Early vigor
		day	number of days from sowing	Effective temperature sum	day	number of days from sowing	Effective temperature sum	
S160	20.04.04	26.VII	97	907,1	26.VII	97	907,1	6
	24.04.06	12.VII	79	857,6	10.VII	77	816,6	6
	19.04.07	8.VII	80	831,3	9.VII	81	841	7
S314B	20.04.04	24.VII	95	883,4	24.VII	95	883,4	7
	24.04.06	11.VII	78	838,8	8.VII	75	780,8	8
	19.04.07	5.VII	77	799,1	4.VII	76	791,1	9
S50676	20.04.04	29.VII	100	942,1	26.VII	97	907,1	7
	24.04.06	23.VII	90	1041,8	13.VII	80	877,9	8
	19.04.07	17.VII	89	953,7	11.VII	83	859,2	8
S63322-3	20.04.04	23.VII	94	869,4	23.VII	94	869,4	9
	24.04.06	15.VII	82	908,8	12.VII	79	857,6	9
	19.04.07	8.VII	80	831,3	8.VII	80	831,3	9
S68911	20.04.04	26.VII	97	907,1	24.VII	95	883,4	9
	24.04.06	16.VII	83	919,9	11.VII	78	838,8	9
	19.04.07	11.VII	83	859,2	10.VII	82	849,2	9
S336A	20.04.04	27.VII	98	917,9	27.VII	98	917,9	6*
	24.04.06	15.VII	82	908,8	9.VII	76	798,8	8
	19.04.07	9.VII	81	841,0	7.VII	79	820,5	9

* 75% loss of plants was noticed at V3 stage, thus the estimation of early vigor at V4 stage was hindered.
