# Supplementary material for: Molecular foundations of chilling-tolerance of modern maize
Source: BMC Genomics. 2016 Feb 20;17:125. doi: 10.1186/s12864-016-2453-4 (PMC4761173; doi:10.1186/s12864-016-2453-4)
Supplement: Additional file 2: — Temperatures at Smolice location in 2004, 2006, and 2007. (PDF 241 kb) [file 12864_2016_2453_MOESM2_ESM.pdf]

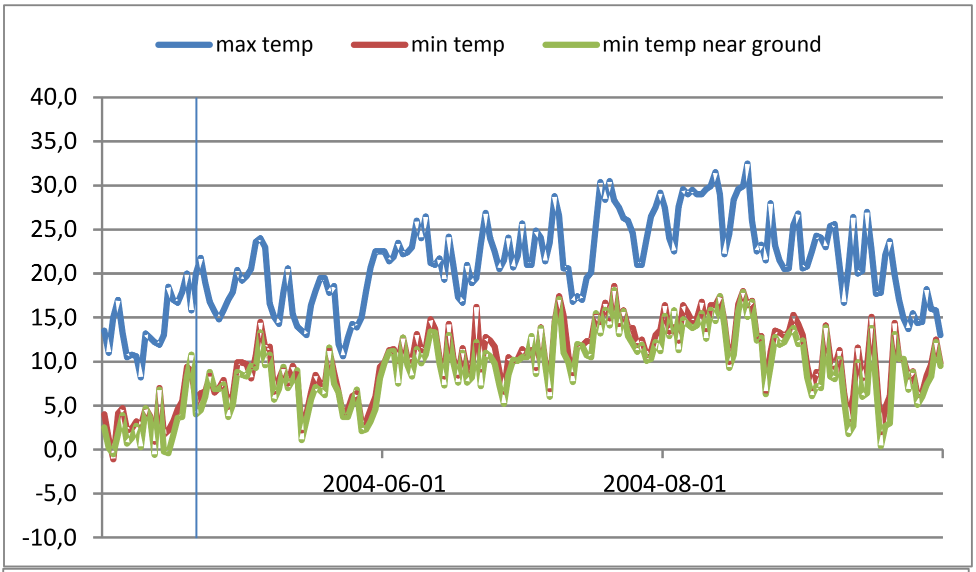

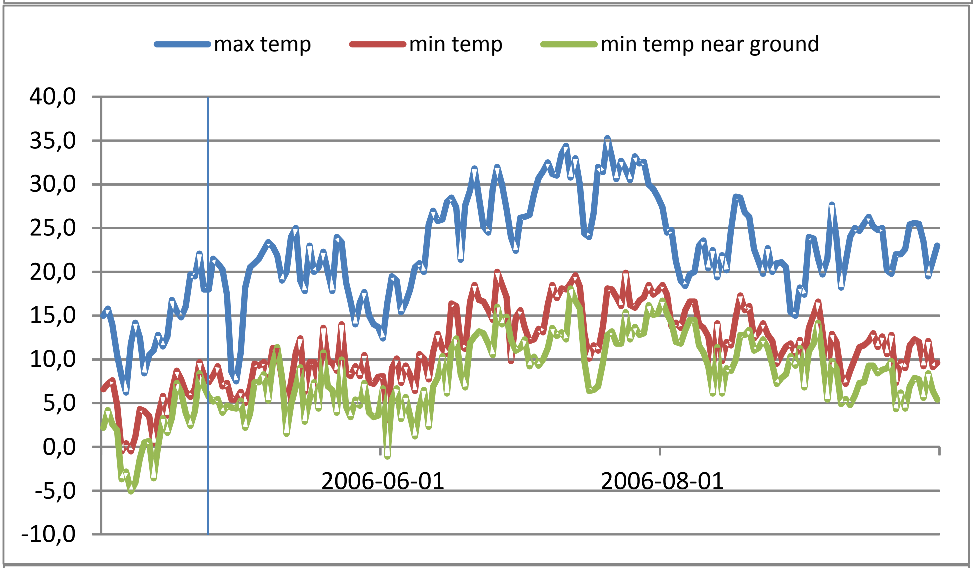

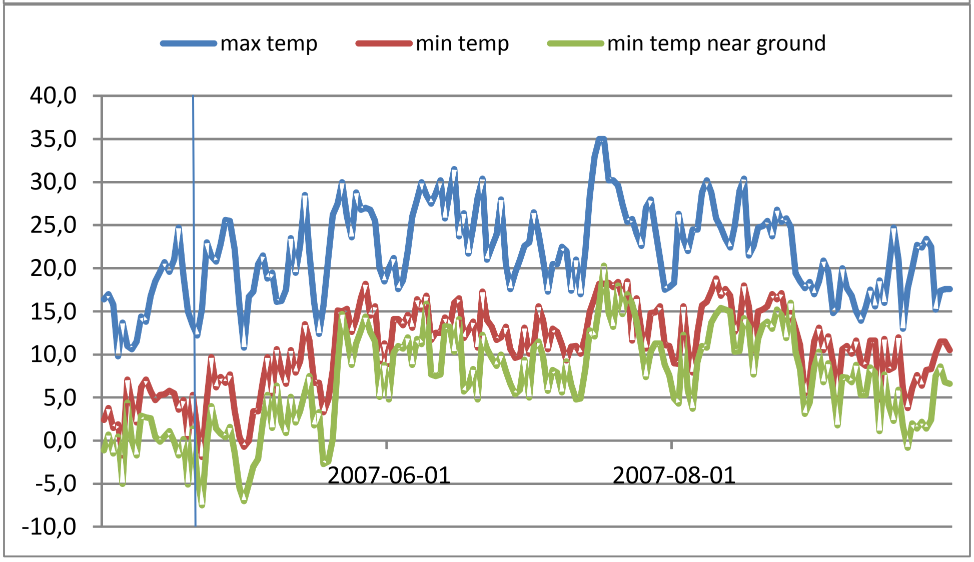

Additional file 2. Air maximum temperatures. minimum temperatures and minimum temperatures near ground [ºC] during April – September periods of 2004, 2006 and 2007 of three inbred lines estimated visually at Smolice (West Poland) location. Vertical lines mark date of sowing: 20.04.2004, 24.04.2006, and 19.04.2007.
