# Supplementary material for: Molecular foundations of chilling-tolerance of modern maize
Source: BMC Genomics. 2016 Feb 20;17:125. doi: 10.1186/s12864-016-2453-4 (PMC4761173; doi:10.1186/s12864-016-2453-4)
Supplement: Additional file 3: — Primers for RT-qPCR. (PDF 219 kb) [file 12864_2016_2453_MOESM3_ESM.pdf]

Additional file 3. Primers for RT-qPCR

**Probe ID**	**Expected product length [bp]**	**Primer sequences**
MZ00023628	80 pz	*Forward:* CTTTTGCTTCCCTTCCTGCTACC<br>*Reverse:* ATCCTGATGATTCTGTCGCCA
MZ00000759	105 pz	*Forward:* CGACATTCTGGCGGGCA<br>*Reverse:* GAGACGACGGCGACGGC
MZ00001512	180 pz	*Forward*: CGCAACATCACCATTCACCC<br>*Reverse:* GACGGCATTACCACGATACACG
MZ00003671	280 pz	*Forward:* GGAGGCGGAGGAGATGCTG<br>*Reverse:* GAGGGTGTGGCTGACGAGG
MZ00013934	290 pz	*Forward:* CCTGAGCACCGCCGATG<br>*Reverse:*: TGGAGACAAGTGGAAGGGAACC
MZ00018865	115 pz	*Forward:* GGGCAAGTCGCAGTCGTTC<br>*Reverse*: CCACATCATCCCTCCCAGTCC
MZ00019074	130 pz	*Forward:* GGAGTAGATACTGGAGGGCGG<br>*Reverse:* AGAAGAATACGGCATCACTTGTTTG
MZ00019555	140 pz	*Forward*: GATGGTGCCCAAGGACGAC<br>*Reverse:* GCGGAAGCCCACGACG
MZ00019753	85 pz	*Forward:* ATTCCTCCCTCTGCTTCGTCA<br>*Reverse:* CGTGGCTGCTGCTGCTG
MZ00020524	100 pz	*Forward:* GCCGCTGCTCAAGGACGA<br>*Reverse:* GAGGTGGTAGGGCTGGAAGAAG
MZ00020668	250 pz	*Forward:* GAGGAGGGCGACGAGGAG<br>*Reverse:* CTGCGGCTGCGGCTG
MZ00021278	105 pz	*Forward:* CTCCGCCGACCCTTCC<br>*Reverse*: GCACAGGCTGGTGACGC
MZ00023547	220 pz	*Forward:* ACTGGTATTGCTCGCTGTCTCG<br>*Reverse:* TCCACATCTCCGCATCTATTGAA
MZ00024268	165 pz	*Forward:* ATAATGATGATGGAGGGAGCGG<br>*Reverse:* CCAGCAAACGAACGAAGCAC
MZ00026192	190 pz	*Forward:* CGCACAAGGTCCTGTTCATCTC<br>*Reverse:* ATCTCTCCCTCGTCGCCG
MZ00026395	90 pz	*Forward:* CGATTTGACGACCCCAGTGC<br>*Reverse:* ATGGCGGATAGGTTCGTGGATG
MZ00026450	120 pz	*Forward*: TGCTATCGGCTCTCAACAACG<br>*Reverse:* TCCACATCCTCCCTCCCACT
MZ00029516	95 pz	*Forward*: GGCAGCCTTCTTCGTCCTCC<br>*Reverse*: GAACGCTACGCTCTCGGGG
MZ00030116	90 pz	*Forward:* TGCGACGCCCAACTAAAAGC<br>*Reverse:* TTGAGGACGGACTTGCCAGG
MZ00030250	150 pz	*Forward:* GCAGGAGATGGTGTTCTTGGC<br>*Reverse:* CTTCAGCAGTTGATGGCAGGTAA
MZ00032781	260 pz	*Forward:* GAATCAAGCAAACCAAGCAGC<br>*Reverse:* CGGAGAAGGAGCGGAGGG
MZ00034417	200 pz	*Forward:*<br>TACAGACTACAGACCACACAGTATCAGC<br>*Reverse:* GAAATGGACCAGAAGAGGGCG

MZ00038695	175 pz	*Forward:* ATGAGTGGAGTCACCTGCTGCC<br>*Reverse*: AAGTAGGACGAGTTCTTGTTCTGC
MZ00040859	180 pz	*Forward*: AACATCCAGGGCATCACGAA<br>*Reverse:* GGTCTTGCGGCGAGCG
MZ00043309	280 pz	*Forward:* GCTGTCTTGCTCGTTTTCGC<br>*Reverse:* AAATGACCATGCCTCGCCA
MZ00044642	210 pz	*Forward:* TGCTGCTGATTGTGTCATTGGA<br>*Reverse:* GAGGTGGAATACGAAGACGGCTA
MZ00050355	100 pz	*Forward:* GCCTCGCCCAACAACACC<br>*Reverse:* CCCACTTCCGTTCCACCAC
MZ00050420	90 pz	*Forward:* GCTCCCGTTCGGCTACTTCC<br>*Reverse:* ACTCGTTCTCGCTGCTGTCG
MZ00056664	120 pz	*Forward:* GGGTGTCTGGCGTGTTCC<br>*Reverse:* GATGTCCTTGTCGGGGTTCT
