# Supplementary material for: Molecular foundations of chilling-tolerance of modern maize
Source: BMC Genomics. 2016 Feb 20;17:125. doi: 10.1186/s12864-016-2453-4 (PMC4761173; doi:10.1186/s12864-016-2453-4)
Supplement: Additional file 4: — RT-qPCR versus microarray results. (PDF 185 kb) [file 12864_2016_2453_MOESM4_ESM.pdf]

Additional file 4. RT-qPCR *versus m*icroarray results. ΔΔCt – change of expression of the gene of interest between cold-treated (c) and control (k) plants estimated by means of RT-qPCR. For RT-qPCR results, data are means of 4 biological experiments (with 3 technical replications) ± SD. For microarray results, *means statistical significance at false discovery rate correction set at 0.05 (p-value = 0.0054); ns –difference not significant.

	**Real-time PCR expression change ΔΔCt**			**Microarrays expression change log_2(c/k)**		
	**S68911**	**S50676**	**S160**	**S68911**	**S50676**	**S160**
MZ00000759	1.05 +-0.26	0.97 +-0.28	1.32 +-0.49	2.23*	1.27ns	1.04ns
MZ00001512	2.17 +-0.04	0.52 +-0.25	0.50 +-0.18	2.48*	0.19ns	0.64ns
MZ00003671	2.54 +-0.55	1.00 +-1.08	0.51 +-0.07	2.9*	2.3*	0.13ns
MZ00013934	0.25 +-1.84	0.54 +-0.23	0.13 +-1.05	1.77*	0.42ns	0.7ns
MZ00016708	1.53 +-0.62	1.54 +-1.07	0.35 +-0.22	1.83*	2.32*	0.16ns
MZ00018865	-2.69 +-0.73	-2.28 +-0.61	-1.25 +-0.65	-2.17*	-2.47*	-1*
MZ00019074	3.39 +-1.3	2.84 +-0.12	0.59 +-0.53	2.22*	2.5*	0.07ns
MZ00019555	2.05 +-0.46	-0.16 +-0.1	-0.48 +-0.09	1.96*	-0.07ns	-0.5ns
MZ00019753	0.95 +-0.98	0.01 +-0.54	0.3 +-0.27	1.26*	0.32ns	0.45ns
MZ00020524	2.01 +-0.79	1.64 +-0.54	-0.61 +-0.27	2.12*	0.65ns	-0.32ns
MZ00020668	0.95 +-0.79	-0.51 +-0.82	0.30 +-0.33	1.7*	-0.68ns	0.31ns
MZ00021278	-1.98 +-0.67	-0.71 +-0.29	-0.63 +-0.05	-2.1*	-0.88ns	-0.8ns
MZ00023547	2.77 +-0.02	1.49 +-0.15	-0.21 +-0.36	3.29*	1.2ns	-0.07ns

MZ00024268	-0.77 +-0.92	0.09 +-0.81	0.29 +-0.16	-0.2ns	0.08ns	0.12ns
MZ00026192	0.48 +-0.47	-0.27 +-0.1	3.78 +-1.02	0.54ns	-0.31ns	1.9*
MZ00026395	-1.64 +-0.15	-0.83 +-0.47	-0.33 +-1.28	-1.63*	-0.69ns	-0.38ns
MZ00026450	2.11 +-0.22	0.47 +-0.39	0.37 +-0.18	2.3*	1.32ns	0.16ns
MZ00029516	-1.26 +-0.45	-1.54 +-2.49	-0.88 +-0.07	-1.37*	-1.5*	-0.47ns
MZ00030116	-1.40 +-1.26	2.85 +-0.32	3.61 +-0.98	0.54ns	2.91*	2.14*
MZ00030250	-0.47 +-0.08	-0.44 +-0.34	1.36 +-0.42	-0.37ns	-0.24ns	1.56*
MZ00032781	-0.07 +-0.52	-0.35 +-0.2	-0.75 +-0.52	-1.85*	-0.58ns	-0.9ns
MZ00034417	0.69 +-0.11	-0.28 +-0.2	1.38 +-0.53	0.79ns	-0.4ns	1.14*
MZ00038695	2.09 +-1.23	0.62 +-0.23	0.33 +-0.13	1.77*	0.45ns	0.06ns
MZ00040859	0.97 +-0.94	0.34 +-0.31	-0.18 +-0.03	2.45*	0.45ns	-0.27ns
MZ00043309	1.32 +-0.5	1.10 +-0.54	0.13 +-0.04	2.36*	1.97*	0.12ns
MZ00044642	-2.61 +-0.54	-1.12 +-0.18	-0.52 +-0.43	-2.57*	-1.2ns	-0.7ns
MZ00050355	-1.95 +-0.38	0.27 +-0.23	-0.36 +-0.21	-1.97*	0.11ns	-0.6ns
MZ00050420	1.41 +-0.44	0.54 +-0.27	0.43 +-0.05	1.79*	0.7ns	0.48ns
MZ00056664	-1.78 +-1.07	0.28 +-0.09	-0.72 +-0.2	-2.32*	0.05ns	-0.68ns
