# Supplementary figures and images for: Molecular foundations of chilling-tolerance of modern maize
Source: BMC Genomics. 2016 Feb 20;17:125. doi: 10.1186/s12864-016-2453-4 (PMC4761173; doi:10.1186/s12864-016-2453-4)

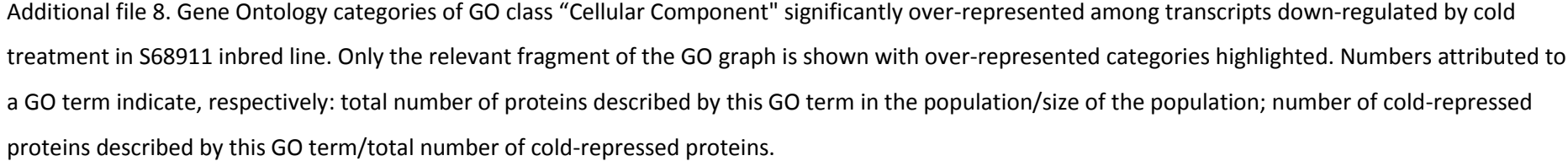

Supplement: Additional file 8: — Gene Ontology categories of GO class “Cellular Component” significantly over-represented among transcripts down-regulated by cold treatment in S68911 inbred line. (PDF 159 kb) [file 12864_2016_2453_MOESM8_ESM.pdf]
