# Supplementary material for: Molecular foundations of chilling-tolerance of modern maize
Source: BMC Genomics. 2016 Feb 20;17:125. doi: 10.1186/s12864-016-2453-4 (PMC4761173; doi:10.1186/s12864-016-2453-4)
Supplement: Additional file 9: — Gene Ontology categories of GO class “Biological Process” significantly over-represented among transcripts up-regulated by cold treatment in S68911 inbred line. (PDF 247 kb) [file 12864_2016_2453_MOESM9_ESM.pdf]

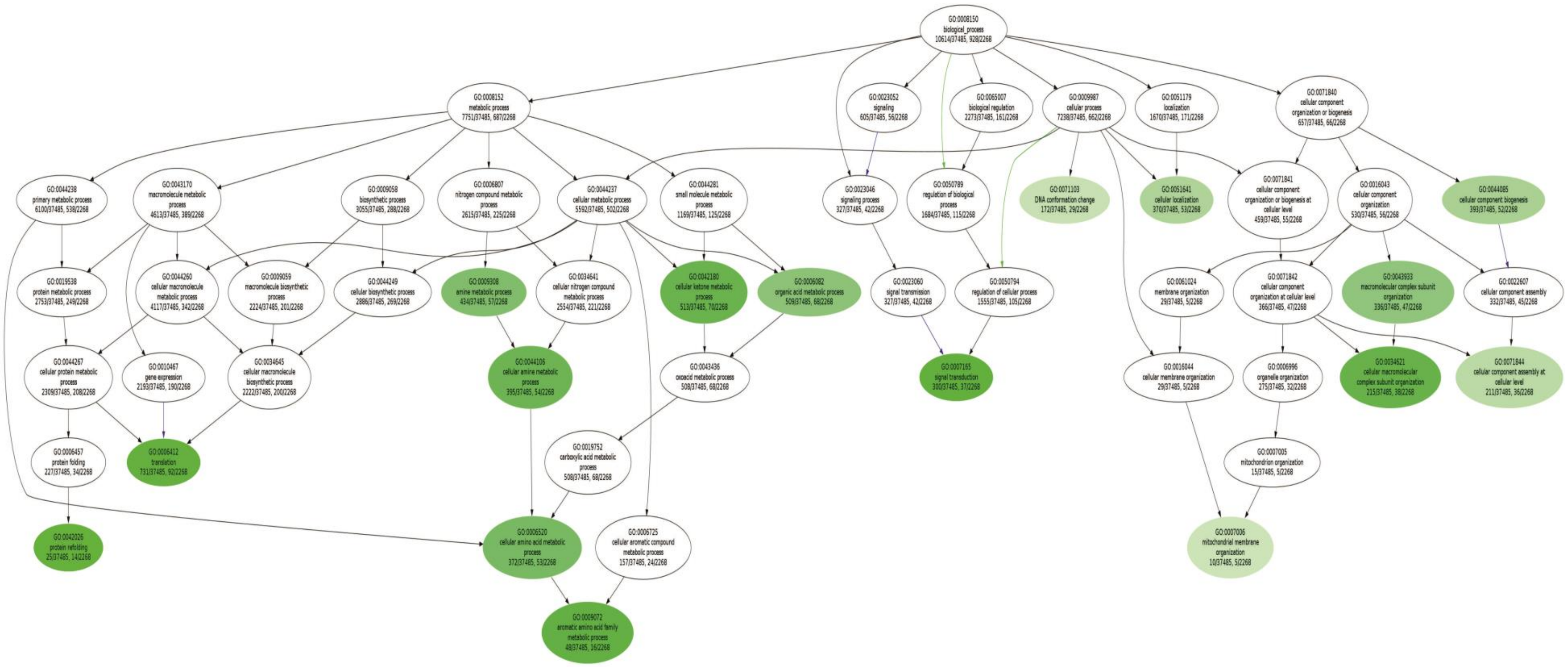

Additional file 9. Gene Ontology categories of GO class “Biological Process" significantly over-represented among transcripts up-regulated by cold treatment in S68911 inbred line. Only the relevant fragment of the GO graph is shown with over-represented categories highlighted. Numbers attributed to a GO term indicate, respectively: total number of proteins described by this GO term in the population/size of the population; number of cold-repressed proteins described by this GO term/total number of cold-repressed proteins.
