# Supplementary material for: Molecular foundations of chilling-tolerance of modern maize
Source: BMC Genomics. 2016 Feb 20;17:125. doi: 10.1186/s12864-016-2453-4 (PMC4761173; doi:10.1186/s12864-016-2453-4)
Supplement: Additional file 25: — Comparison of nucleotide sequences of putative promoter region of GRMZM2G331566 gene. (PDF 246 kb) [file 12864_2016_2453_MOESM25_ESM.pdf]

Additional file 25. Comparison of nucleotide sequences of putative promoter region of GRMZM2G331566 gene represented by microarray probe MZ00026395 in diverse maize lines

Position[1]	Inbred line			
	**S68911**	**S50676**	**S160**	**B73**
-739	C	A	A	C
-701	-	GCTGTGG	GCTGTGG	-
-646	G	C	C	G
-575	A	T	T	A
-546	-	TCGCCGCCTT GAGAAACCAA AGCC	TCGCCGCCTT GAGAAACCAA AGCC	TCGCCGCCTT GAGAAACCAA AGCC
-501	CAACCA	CAACCA	CAACCA	-
-490	C	A	A	A
-437	C	T	T	T
-417	-	C	C	C
-414	-	C	C	-
-392	C	-	-	C
-390	-	TA	TA	-
-380	A	C	C	A
-378	A	G	G	A
-373	* 132 bp	-	-	-
-316	-	GT	GT	GT
-204	C	T	T	C
-193	C	T	T	C
-99	A	C	C	C
-43	-	C	C	-
-39	ACCACTG	-	-	-
-17	A	A	A	T
+18	T	C	C	C
+22	T	C	C	T
+102	T	C	C	C
+105	G	G	G	A
+192	C	A	A	C

* GGGCCTGTTTGGTTCGTGGCTAACTATGCCACACTTTGCCTAAGGTTAGTCG TTCGAATTAAAGAACTAACCTTAAGCAGAAAAGTTAGGCAAAGTGTGGCAA TTGAGGCTGCGATCCAAACATACCCCCTG

[1]position is given according to B73 sequence. relative to A in AUG translation start codon numbered +1
