# Supplementary material for: Molecular foundations of chilling-tolerance of modern maize
Source: BMC Genomics. 2016 Feb 20;17:125. doi: 10.1186/s12864-016-2453-4 (PMC4761173; doi:10.1186/s12864-016-2453-4)
Supplement: Additional file 26: — Primers for miRNA. (PDF 8 kb) [file 12864_2016_2453_MOESM26_ESM.pdf]

Additional file 26. Primers for miRNA

	Forward primers	Primers for reverse transcription
zma-MIR164 a/b/c/d/g*	CATGCATGGAGAAGCAGGGCA	GTCGTATCCAGTGCAGGGTCCGAGGTATTCGC ACTGGATACGACTGCACG
zma-MIR167 a/b/c/d*	GCACGGTGAAGCTGCCAGCAT	GTCGTATCCAGTGCAGGGTCCGAGGTATTCGC ACTGGATACGACTAGATC
zma-MIR172 b/c/d*	GCTCCGAGAATCTTGATGAT	GTCGTATCCAGTGCAGGGTCCGAGGTATTCGC ACTGGATACGACTGCAGC
zma-MIR168 a/b*	CGACTGTCGCTTGGTGCAGAT	GTCGTATCCAGTGCAGGGTCCGAGGTATTCGC ACTGGATACGACGTCCCG
zma-MIR171 a/d/e/i/j/n	TGTTCGTGATTGAGCCGCGCC	GTCGTATCCAGTGCAGGGTCCGAGGTATTCGC ACTGGATACGACGATATT
zma-MIR162	GCTCCGTCGATAAACCTCTG	GTCGTATCCAGTGCAGGGTCCGAGGTATTCGC ACTGGATACGACTGGATG
zma-MIR169 a/b	TGTCCGCAGCCAAGGATGACT	GTCGTATCCAGTGCAGGGTCCGAGGTATTCGC ACTGGATACGACTCGGCA
Universal reverse primer*	GTGCAGGGTCCGAGGT	

*according to Liu et al. 2012 [75]
